# Infection-triggered encephalopathy syndromes: a meta-analysis of clinical characteristics and outcomes in 1946 cases

**DOI:** 10.1016/j.eclinm.2026.104163

**Published:** 2026-08-26

**Authors:** Michael Eyre, Velda X. Han, Terrence Thomas, Hiroshi Sakuma, Shekeeb S. Mohammad, Hannah F. Jones, Takayuki Mori, Go Kawano, Vanessa W. Lee, Stephen Malone, Carly Debinski, Hiroya Nishida, Margherita Nosadini, Ming Lim, Russell C. Dale

**Affiliations:** a Department of Imaging Physics & Engineering and Department of Early Life Imaging, School of Biomedical Engineering and Imaging Sciences, King's College London, London, United Kingdom; b Children's Neurosciences, Evelina London Children's Hospital at Guy's and St Thomas' NHS Foundation Trust, London, United Kingdom; c Khoo Teck Puat-National University Children's Medical Institute, National University Health System, Singapore; d Department of Paediatrics, Yong Loo Lin School of Medicine, National University of Singapore, Singapore; e Department of Paediatrics, Neurology Service, KK Women's and Children's Hospital, Singapore; f Department of Brain & Neurosciences, Tokyo Metropolitan Institute of Medical Science, Tokyo, Japan; g Kids Neuroscience Centre, The Children's Hospital at Westmead, Faculty of Medicine and Health, University of Sydney, Westmead, New South Wales, Australia; h Department of Neuroservices, Starship Children's Hospital, Auckland, New Zealand; i Department of Paediatrics, The University of Tokyo Hospital, Tokyo, Japan; j Department of Paediatrics, St Mary's Hospital, Fukuoka, Japan; k Children's Neurosciences, Evelina London Children's Hospital, London, United Kingdom; l Pediatric Neurology Unit, Hospital Tunku Azizah Kuala Lumpur, Malaysia; m Neuroscience Department, Queensland Children's Hospital, South Brisbane, Queensland, Australia; n Monash Children's Hospital, Melbourne, Victoria, Australia; o Paediatric Neurology and Neurophysiology Unit, Department of Women's and Children's Health, University Hospital of Padova, Padova, Italy; p Neuroimmunology Group, Paediatric Research Institute ‘Città della Speranza’, Padova, Italy; q Faculty of Life Sciences and Medicine, King's College London, London, United Kingdom; r Department of Neurology, Hunan Children's Hospital, Changsha, China

**Keywords:** Acute encephalopathy, Febrile encephalopathy, Neuroimaging, Therapeutics, Immunotherapy

## Abstract

**Background:**

Infection-triggered encephalopathy syndromes (ITES) are acute, para-infectious disorders that can cause disability or death. Recognition is increasingly important in viral pandemics, but clinical features and outcomes remain poorly understood.

**Methods:**

PubMed search (inception to 6 March 2024) identified studies with published individual patient data (raw data were not collected from authors). The search was updated on 14 May 2026 using the same terms to identify reports and cases published after the data extraction cutoff. Data were extracted by paediatric neurologists using a standardised proforma. Major syndromes included acute encephalopathy with biphasic seizures and late reduced diffusion (AESD), acute necrotising encephalopathy (ANE), acute shock with encephalopathy and multiorgan failure (ASEM), hemiconvulsion-hemiplegia-epilepsy syndrome (HHE), febrile infection–related epilepsy syndrome (FIRES) and mild encephalopathy with reversible splenial lesion (MERS). We performed a pooled analysis of individual-level published data investigating patient characteristics, infections, and clinical outcomes measured by six-month modified Rankin Scale (mRS). Infection-syndrome associations were analysed with chi-square normalised residuals.

**Findings:**

1946 patients from 656 studies (by ascending age of onset) included 164 ASEM (median age 0.78 years), 217 AESD (1.3 years), 95 HHE (2 years), 414 ANE (3.4 years), 562 FIRES (9 years), and 422 MERS (9.25 years). An updated search identified 658 cases from 171 studies that could be eligible for inclusion. AESD was linked to human herpesvirus 6 (z = 12.87); ANE with influenza A (z = 11.1), SARS-CoV-2 (z = 9.1) and influenza B (z = 4.3); MERS with rotavirus infection (z = 7.48); and FIRES with absence of microbiological identification (z = 18.2) (all p < 0.001).

Neuroimaging was often delayed. Magnetic resonance imaging (MRI) brain restricted diffusion was the commonest finding, typically bilateral (except HHE). Characteristic patterns included thalamic involvement with or without haemorrhagic changes in ANE, corpus callosum involvement in MERS, and subcortical white matter involvement in AESD and HHE. Cerebrospinal fluid pleocytosis occurred mostly in FIRES and MERS, and elevated protein in ANE.

Immunotherapy (commonly steroids, immunoglobulin) including biologics (anakinra, tocilizumab) was used most in ANE and FIRES. Acute mortality was 12% (227/1946), highest in ASEM (50%) and ANE (35%). There was good six-month outcome (mRS 0–2) in 52% (95% CI 50–55; 615/1174): MERS 99% (95% CI 97–100; 323/326), AESD 54% (95% CI 44–64; 50/93), FIRES 38% (95% CI 33–44; 120/314), ANE 33% (95% CI 27–38; 88/269), HHE 16% (95% CI 7–32; 5/32), and ASEM 15% (95% CI 9–24; 14/91).

**Interpretation:**

ITES showed distinct demographic, clinico-radiological features and outcomes. This largest ITES cohort to date highlights the importance of timely imaging, intervention, and future global collaboration to advance diagnosis, treatment, and pandemic preparedness.

**Funding:**

No funding was received for this work.

**Research in context**

**Evidence before this study**

A recent international consensus has unified infection-triggered encephalopathy syndromes (ITES), defining five core syndromes and two related conditions. These rare clinico-radiological syndromes are characterised by para-infectious, hyperacute onset with outcomes ranging from complete recovery to mortality. There is increasing evidence that the pathogenesis of ITES involves systemic and central nervous system inflammation driven by a dysregulated cytokine response (‘cytokine storm’), excitotoxicity, and cerebral oedema. Clinical characteristics, treatment approaches, and outcomes have been reported mainly in case reports and small cohorts. No systematic review or meta-analysis comparing these syndromes was identified, highlighting the lack of comprehensive synthesised evidence prior to this study.

We conducted a PubMed search (inception to 6 March 2024) for previously published studies on ITES using keywords including acute encephalopathy with biphasic seizures and late reduced diffusion (AESD); acute infantile encephalopathy predominantly affecting the frontal lobes (AIEF); acute leukoencephalopathy with restricted diffusion (ALERD); acute fulminant cerebral oedema (AFCE); acute necrotising encephalopathy (ANE), acute haemorrhagic necrotising encephalopathy (AHNE); febrile infection–related epilepsy syndrome (FIRES); new-onset refractory status epilepticus (NORSE); devastating epilepsy in school-aged children (DESC); acute encephalitis with refractory repetitive partial seizures (AERRPS); hemiconvulsion–hemiplegia–epilepsy syndrome (HHE); hemorrhagic shock and encephalopathy syndrome (HSES); and reversible splenial lesion syndrome (RESLES), including mild encephalopathy with a reversible splenial lesion. We included relevant spelling variants and related terminology to maximise sensitivity. Studies with individual-level published data in Chinese, English, French, Italian, Japanese, Portuguese or Spanish were included. A pooled narrative analysis was conducted, including 1946 patients from 656 studies across six primary ITES syndromes. An updated search on 14 May 2026 identified 658 cases from 171 studies that could be eligible for inclusion.

**Added value of this study**

To the best of our knowledge, this meta-analysis of pooled individual-level published data collects the largest ITES cohort to date. We report micro-organisms detected, demographic, clinical and neuroimaging features and outcomes of each syndrome.

**Implications of all the available evidence**

This meta-analysis aims to inform clinicians on ITES and enhance clinical recognition, timely intervention, and the need for future coordinated international initiatives to advance diagnostic and therapeutic strategies for these devastating syndromes.

## Introduction

Infection-triggered encephalopathy syndromes (ITES) are parainfectious disorders with febrile encephalopathy, mainly affecting children, that can cause severe disability or death.^1^ They present with rapidly evolving encephalopathy, seizures, and characteristic brain imaging changes, typically within seven days of a systemic infection.^1^ Unlike infectious encephalitis, there is no direct brain infection, and unlike autoimmune encephalitis, no adaptive autoimmune processes are involved.^1^ Although the exact mechanism for ITES is currently unclear, emerging evidence suggests an excessive systemic inflammatory and glial response (compatible with a ‘cytokine storm’) causes excitotoxic brain injury, sometimes linked to genetic susceptibility.^1^, ^2^, ^3^, ^4^, ^5^

Our group recently led an international consensus initiative to standardise definitions based on clinical and neuroimaging features,^1^ proposing five core clinical syndromes and two related entities (Table 1). The five core syndromes are: (1) acute encephalopathy with biphasic seizures and late reduced diffusion (AESD), considered as part of a spectrum including acute infantile encephalopathy predominantly affecting the frontal lobes (AIEF),^7^ and acute leukoencephalopathy with restricted diffusion (ALERD) ^8^^,^^9^; (2) acute necrotising encephalopathy (ANE), with acute haemorrhagic necrotising encephalopathy (AHNE) considered a subgroup ^10^; (3) mild encephalopathy with a reversible splenial lesion (MERS); (4) acute fulminant cerebral oedema (AFCE); and (5) acute shock with encephalopathy and multiorgan failure (ASEM) (previously known as haemorrhagic shock and encephalopathy syndrome [HSES]). Additionally, two syndromes, febrile infection–related epilepsy syndrome (FIRES)^6^^,^^11^ and hemiconvulsion–hemiplegia–epilepsy (HHE) syndrome,^12^ were classified as related entities, reflecting growing recognition of immune-mediated mechanisms in their pathogenesis.^2^^,^^11^

**Table 1:** Clinical definitions of five infection-triggered encephalopathy syndromes (ITES) and two related entities.

**Core definition of definite infection-triggered encephalopathy syndrome (ITES)**^1^ 1 The mode of onset Essential: a previous febrile illness starting within a week before the onset of the first neurological manifestation. Common: fever is typically still present at neurological onset. 2 Clinical Essential: a clinical presentation of decreased or altered level of consciousness, altered mental status, lethargy, or personality change, lasting for >24 h. Common: these symptoms may not last for >24 h in rapidly progressive fatal cases or mild encephalopathy with a reversible splenial lesion. 3 Neuroradiological and other investigation Essential: neuroradiological abnormalities specific to each syndrome. 4 Diseases to be excluded (refer to Sakuma et al.^1^)
**Diagnostic criteria of acute fulminant cerebral oedema (AFCE) and acute shock with encephalopathy and multiorgan failure (ASEM)**^1^ 1 The mode of onset Essential: febrile illness preceding or concurrent to the onset of neurological manifestations. 2 Clinical Essential: acute encephalopathy: rapid reduction of consciousness and/or seizures. Essential (for ASEM): shock not due to massive haemorrhage or cardiac disease. 3 Neuroradiological and other investigation Essential: progression to diffuse cerebral oedema evident on neuroimaging and/or autopsy. Common: appearing within 48 h of onset if imaged, although rapidly progressive fatal cases may not fulfil these criteria before reaching an irreversible state. Essential (for ASEM): signs of multiple organ failure (at least three of the following): (1) anaemia; (2) thrombocytopaenia; (3) disseminated intravascular coagulation; (4) acidosis; (5) raised hepatocellular enzymes; (6) renal dysfunction. 4 Diseases to be excluded (refer to Sakuma et al.^1^)
**Diagnostic criteria of definite acute encephalopathy with biphasic seizures and late reduced diffusion (AESD)**^1^ 1 The mode of onset Essential: febrile illness preceding or concurrent to the onset of neurological manifestation. 2 Clinical Essential: a clinical presentation of decreased or altered level of consciousness, altered mental status, lethargy, or personality change, lasting for >24 h. Common: • Early seizure(s) associated with the fever on days 1–2, usually lasting longer than 30 min. • Late seizures on day 3–7, most often in a cluster of focal seizures, with interictal encephalopathy (deterioration of consciousness level). • In severe cases, transient recovery after early seizures is not evident, and therefore early and late seizures may not be distinguished. Late seizures may also be masked by aggressive antiseizure therapy. 3 Neuroradiological and other investigation Essential: restricted diffusion on MRI in the subcortical white matter (bright tree appearance- BTA) at day 3–14 after onset of neurological symptoms. Common: • Restricted diffusion on MRI in the cortex. • Spared (unaffected) pre- and postcentral gyrus (central sparing). 4 Diseases to be excluded (refer to Sakuma et al.^1^)
**Diagnostic criteria of acute necrotising encephalopathy (ANE)**^1^ 1 The mode of onset Essential: febrile illness preceding or concurrent to the onset of neurological manifestations. 2 Clinical Essential: a clinical presentation of decreased or altered level of consciousness, altered mental status, lethargy, or personality change, lasting for >24 h. Common: rapid (hours) reduction of consciousness with or without seizures and/or focal neurological signs (pyramidal or extrapyramidal motor signs). 3 Neuroradiological and other investigation Essential: symmetrical thalamic lesions on head computed tomography and/or MRI Common: • Thalamic lesions are typically not homogeneous and often show concentric structures, suggesting necrosis and/or haemorrhage in the centre. • Symmetrical and multiple brain lesions in the periventricular white matter, internal capsules, putamina, temporal lobes, upper brainstem tegmentum, or cerebellum. • Cerebrospinal fluid examination typically shows normal cell counts and increased protein concentration. • Elevated serum transaminase levels with no elevation in serum ammonia levels. 4 Diseases to be excluded (refer to Sakuma et al.^1^)
**Diagnostic criteria of mild encephalopathy with a reversible splenial lesion**^1^ 1 The mode of onset Essential: febrile illness preceding or concurrent to the onset of neurological manifestations. 2 Clinical Essential: a clinical presentation of decreased or altered level of consciousness, altered mental status, lethargy, or personality change, lasting for >24 h. Common: • Other clinical features include seizures or hallucinations. • Self-limiting clinical course with spontaneous recovery. • Alteration in consciousness and change in personality/behaviour may be intermittent and may occasionally not persist past 24 h. 3 Neuroradiological and other investigation Essential: a splenial corpus callosum lesion with homogeneously restricted diffusion on MRI. Common: • Lesion involving at least the splenium. It may expand to the entire corpus callosum or involve the cerebral white matter symmetrically. • The corpus callosum lesion usually shows hyperintensity on T2-weighted images/FLAIR, and less commonly hypointensity on T1-weighted images. • If interval imaging is performed, the lesion is typically resolved within 1–2 months. 4 Diseases to be excluded (refer to Sakuma et al.^1^)
**ITES-related entities**
**Diagnostic criteria of febrile infection–related epilepsy syndrome (FIRES)**^6^ New-onset refractory status epilepticus (NORSE) is a clinical presentation, not a specific diagnosis, in a patient without active epilepsy or other preexisting relevant neurological disorder, with new onset of refractory status epilepticus without a clear acute or active structural, toxic or metabolic cause. FIRES is a subcategory of NORSE, applicable for all ages, that requires a prior febrile infection starting between 2 weeks and 24 h prior to onset of refractory status epilepticus, with or without fever at onset of status epilepticus.
**Diagnostic criteria of hemiconvulsion-hemiplegia-epilepsy syndrome (HHE)**^6^ Hemiconvulsion-hemiplegia and epilepsy syndrome is a specific syndrome in a patient <2 years old, presenting as NORSE with unilateral motor seizures, high- grade fever at the time of onset of refractory status epilepticus, and unilaterally abnormal acute imaging, followed by hemiparesis lasting at least 24 h, and excluding definite infectious encephalitis.

MRI = magnetic resonance imaging, FLAIR = fluid-attenuated inversion recovery.

As these disorders are rare, available studies mostly comprise case reports and small series with no meta-analysis directly comparing syndromes, leaving differences in clinical features, treatments, and outcomes unclear. We performed a pooled meta-analysis of individual-level published cases, with four primary objectives: (1) to characterise patient demographics and clinico-radiological features; (2) to identify markers of disease severity; (3) to examine patterns of immunotherapy use; and (4) to compare clinical outcomes, across the ITES syndromes. We aim to raise awareness of these syndromes and support future research and treatment frameworks for clinicians.

## Methods

### Systematic search

**Fig. 1:**
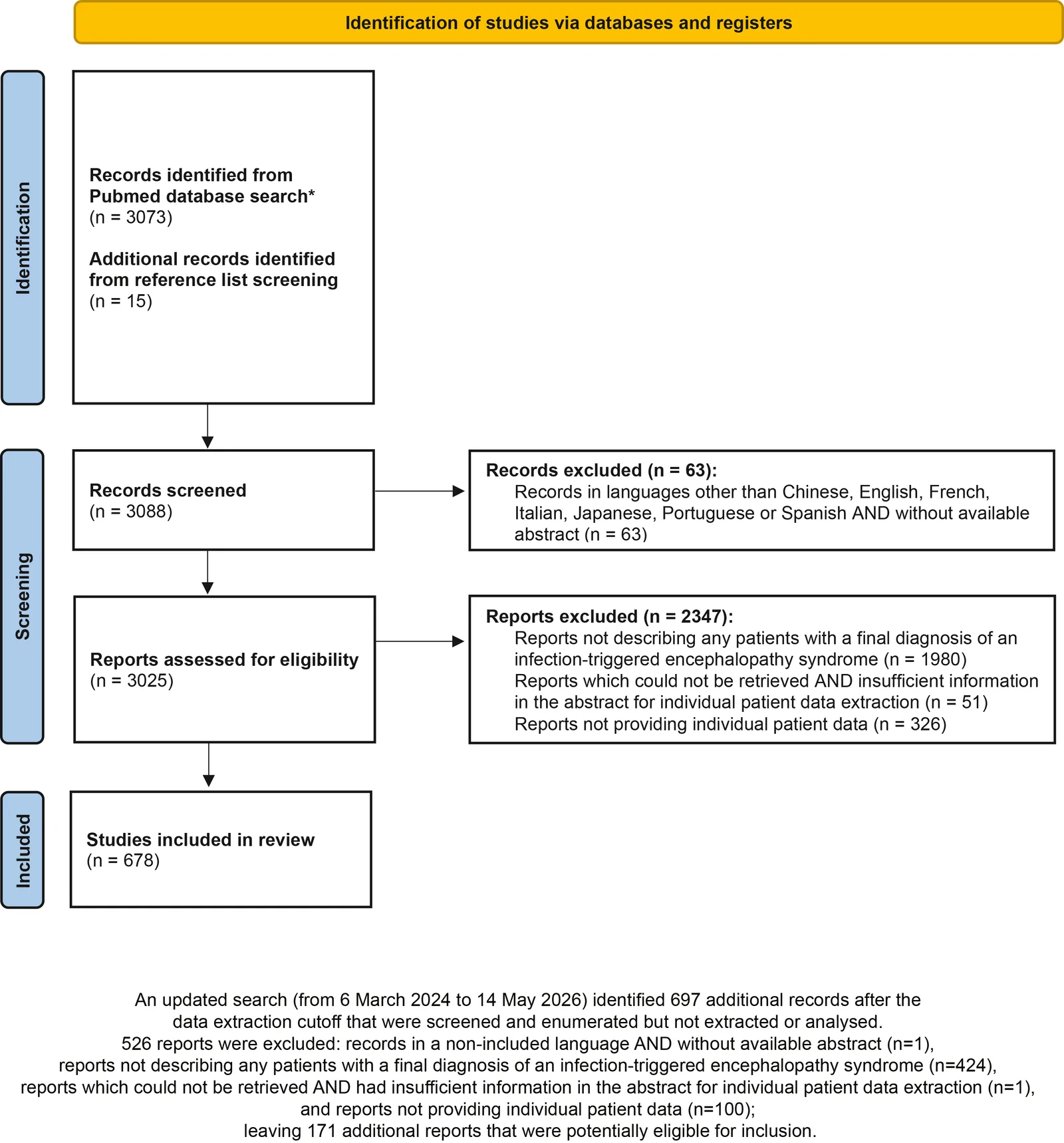
Study profile. Records were identified through PubMed search (up to 6 March 2024) (n = 3073) and additional sources (n = 15). Following removal of duplicates, full-text articles were assessed for eligibility, and studies not meeting inclusion criteria were excluded with reasons (n = 2347). A total of 678 studies were included in the analysis of pooled individual-level published data.

This study followed Preferred Reporting Items for Systematic Reviews and Meta-Analyses (PRISMA) guidelines. PubMed was searched from inception to 6 March 2024 (ME), using terms (Fig. 1, Supplementary Figure S1A) targeting ten syndromes and synonyms: the five core ITES syndromes (AESD, ANE, MERS, AFCE, ASEM) and two related syndromes (FIRES, HHE) as above,^1^ plus three additional ITES subgroups: AIEF,^7^ ALERD,^8^ and AHNE.^10^ Syndromes with ≥50 patients were included in subgroup comparisons (Supplementary Figure S1B).

#### Search terms:

((acute encephalopathy with biphasic seizures and late reduced diffusion) OR (AESD) OR (acute infantile encephalopathy predominantly affecting the frontal lobes) OR (AIEF) OR (acute leukoencephalopathy with restricted diffusion) OR (ALERD)) OR ((acute fulminant cerebral edema) OR (acute fulminant cerebral oedema) OR (AFCE)) OR ((acute necrotizing encephalopathy) AND (ANE)) OR (RANBP2) OR ((acute hemorrhagic necrotizing encephalopathy) AND (covid OR covid-19 OR coronavirus OR SARS‑CoV‑2)) OR ((acute haemorrhagic necrotizing encephalopathy) AND (covid OR covid-19 OR coronavirus OR SARS‑CoV‑2)) OR ((febrile infection related epilepsy syndrome) OR (NORSE) OR (new onset refractory status epilepticus) OR (devastating epilepsy in school aged children) OR (DESC) OR (acute encephalitis with refractory repetitive partial seizures) OR (AERRPS)) OR (hemiplegia hemiconvulsion epilepsy) OR ((hemorrhagic shock and encephalopathy syndrome) OR (haemorrhagic shock and encephalopathy syndrome)) OR ((reversible splenial lesion syndrome) OR (RESLES) OR (mild encephalopathy with reversible splenial lesion)).

#### Inclusion criteria:

(1) Studies in Chinese, English, French, Italian, Japanese, Portuguese or Spanish were eligible if they reported individual-level published data. The minimum required information was the individual's age category (paediatric or adult).
(2) Patients with a final diagnosis of one of the ten syndromes specified above, defined by ITES consensus guidelines^1^ (including previous diagnostic terms,^1^^,^^6^, ^7^, ^8^^,^^10^^,^^12^, ^13^, ^14^, ^15^, ^16^, ^17^ outlined in Supplementary Figure S1A).
(3) The ITES episode was triggered by or associated with infection or febrile illness (regardless of microbiological identification).

#### Exclusion criteria:

(1) Studies providing only grouped (non-individualised) patient data.
(2) Patients for whom the authors did not provide a clear final diagnosis.
(3) Patients with positive anti-neuronal antibodies (e.g., N-methyl-d-aspartate receptor (NMDAR) or gamma-aminobutyric acid type A (GABA-A) receptor antibody in FIRES).

Published individual-level patient data and not raw data were used in this study. Individual authors of studies were not contacted for raw data. Abstracts were included if adequate individual patient data was available.

### Data extraction and harmonisation

Articles identified by the search were screened against the prespecified inclusion criteria by the author group of fifteen paediatric neurologists (each with at least 5 years clinical experience in paediatric neurology). Articles were allocated among team members for screening, as dual independent screening of each article was not feasible at this scale. The paediatric neurologists ensured consistency between diagnoses and descriptions; and used a standardised proforma (Supplementary Excel S1) to extract data for each individual patient.

During data harmonisation, individual patient data entered into proformas were labelled and merged into a master dataset with one row per patient. Completing a second independent double-entry within a realistic timeframe was not feasible, thus central data verification steps were undertaken by author ME (Supplementary Methods). To prevent duplicate entries, author names and patient demographics (age and sex) were cross-checked computationally to identify repeated records. Ambiguities or borderline eligibility for inclusion were not decided by a single rater but were resolved during bi-monthly meetings to ensure data consistency. A final central review validated exclusion of patients without diagnosis of a specific ITES syndrome (ME, RD) (Supplementary Figure S1B).

In order to enumerate reports and cases published after the data extraction cutoff date, the search was updated on 14 May 2026 (ME) with the same terms. Given the scale of the extraction and central review, and to preserve a fixed analytic dataset, the updated search of 14 May 2026 was conducted for enumeration only; additional reports and cases were screened and counted (Fig. 1 and Supplementary Figure S1B footnotes) but not extracted or incorporated into the analysis.

### Study datapoints and definitions

The dataset comprised nine publication-level fields, 630 patient-level fields and 134 MRI fields (up to three timepoints), covering demographics, symptoms, investigations, treatments, outcomes, and long-term sequelae (detailed in Supplementary Methods).

Functional outcome was assessed using the modified Rankin Scale (mRS) at follow-up. If not reported in the original article, mRS was assigned by rater based on clinical data. Outcomes were categorised as follows: mRS 0–2 at any follow-up time was classified as good outcome, under the assumption that functional independence once achieved was maintained. mRS 3–5 was classified as poor outcome only if assessed six months or later from disease onset; patients with mRS 3–5 at final follow-up before six months were not included in outcome data. Death (mRS 6) was classified as poor outcome regardless of follow-up duration.

### Inter-rater agreement

Reliability of overall data extraction and retrospectively assigned mRS was assessed post-hoc in a subset of cases by independent blinded double-rating. For each field we first compared whether both raters assigned a value rather than leaving it blank (scorability), then quantified agreement among co-scored fields using raw agreement, Cohen's κ (quadratic-weighted κ for ordinal data, prevalence-adjusted bias-adjusted κ [PABAK] for categorical data) and intraclass correlation coefficient (ICC) for continuous data, calculated using *statsmodels* (v0.13.5) in Python (v3.10.12). For overall data extraction, 20 cases were re-extracted by a blinded second rater; across 503 patient-level fields (10,060 pairs of observations) mean inter-rater agreement on scorability was 89%, mean raw agreement in co-scored categorical fields was 92% (κ 0.61, fair-to-good) and in ordinal fields was 94% (κ 0.89, excellent), and mean ICC in continuous fields was 0.82. For retrospectively assigned mRS, six raters independently re-scored 20 separate cases each, drawn from those contributing to the six-month outcome analysis, blinded to the original scores (120 cases; 240 paired acute and follow-up ratings). Inter-rater agreement on scorability was 92% (220/240); in 212 dual-rated scores, agreement was excellent for follow-up mRS (κ 0.89, 95% CI 0.80–0.97; n = 115) and fair-to-good for acute-phase mRS (κ 0.58, 95% CI 0.38–0.77; n = 97). Further details are provided in the Supplementary Methods.

### Statistical analyses

This study is a descriptive pooled analysis of individual-level published data from a large number of reports, predominantly single-patient case reports and small series, summarised by syndrome (Supplementary Figure S1C). Because we analysed individual patient data rather than pooling study-level effect estimates, formal methods for assessing publication bias (e.g., funnel plot and Egger's test), between-study heterogeneity (e.g., I^2^ statistic), risk of bias (e.g., RoB-2, RoB-NMA), and certainty of evidence (e.g., GRADE, CINeMA) were not applicable. These instruments are primarily designed to appraise and combine study-level effect estimates from comparative studies, whereas most included studies were single-patient case reports or small series without a comparison group or definable effect estimate.

Given the retrospective nature of individual patient-level data extraction from published reports, missing data were anticipated and varied widely. An available-case analysis was performed, including only variables explicitly reported in the individual reports, with no imputation of missing data. In subgroup analyses, proportions used the total number of patients within each subgroup as the default denominator; denominators are explicitly stated in the results only when they differed from the subgroup total due to clinically significant missing or unavailable data. All data points are provided in Supplementary Excel S2.

Infection-syndrome associations were assessed using chi-square tests with standardised residuals and false discovery rate (FDR)-adjusted p-values (Benjamini–Hochberg) using *stats* in R (v4.5.2). Significance threshold was z-score ≥3.29 or ≤-3.29 (two-sided *p* < 0.001). For key outcomes, we used the Wilson score method to calculate 95% confidence intervals for proportions (post-hoc), using *statsmodels* (v0.13.5) in Python (v.3.10.12). Bar charts and dot plots were generated using *ggplot2* (v4.0.2) in R. Data visualisation and enrichment analyses were performed following standard workflows within the *ggplot2* package. Longitudinal patient status plots (intensive care unit [ICU] admission, survival outside ICU or death) were generated using *matplotlib* (v.3.7.0) in Python. Brain region images were generated by layering custom anatomical masks on a *Biorender* template using *PIL* (v9.3.0) in Python.

### Ethics

There was no requirement for ethics approval or written informed consent, as we used published data that were publicly available.

### Role of the funding source

No funding was received for this work.

## Results

**Fig. 2:**
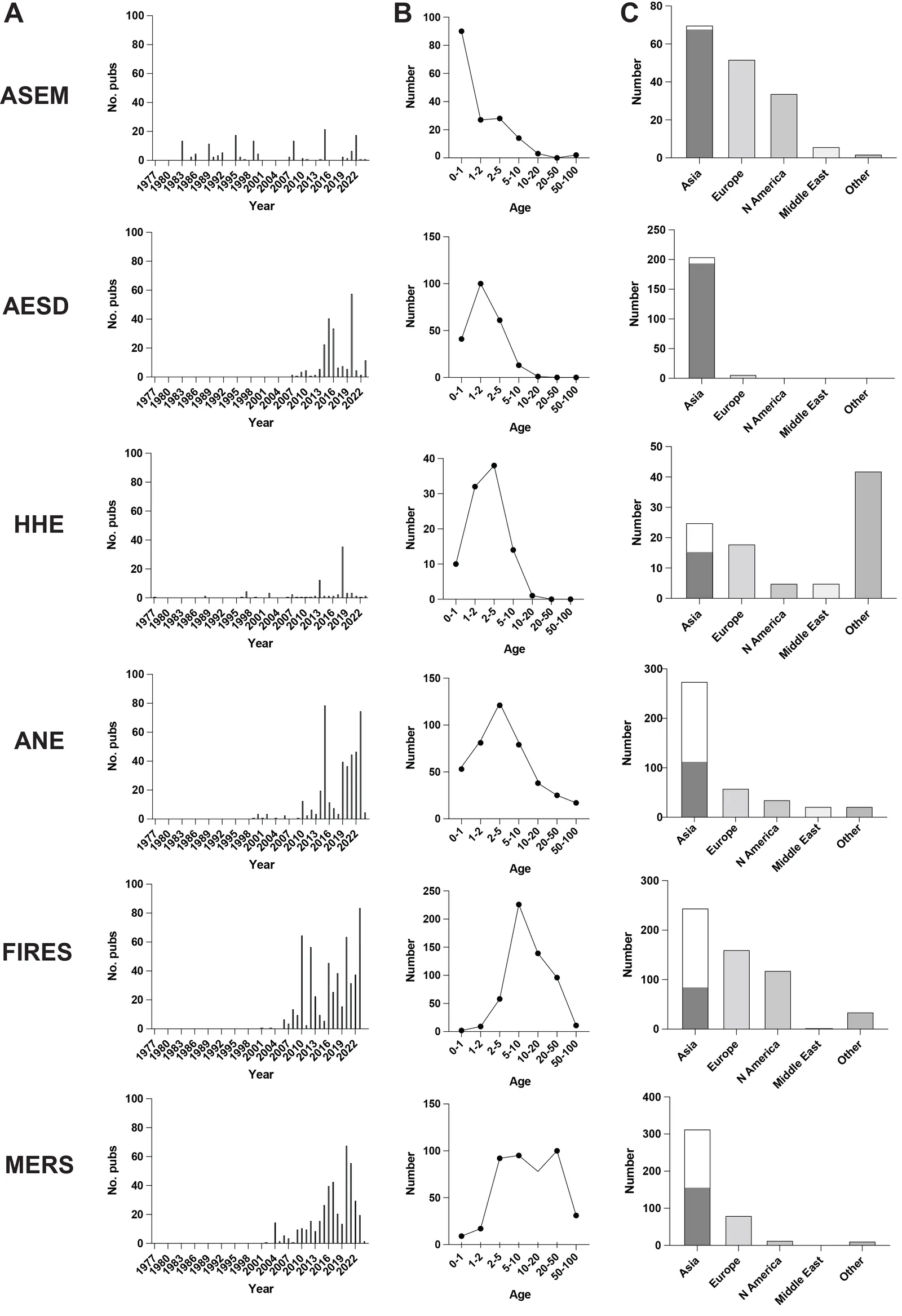
Age at onset and publication characteristics in the six major infection-triggered encephalopathy syndromes (ITES). Publication year (A), patient age at first ITES onset (B), and publication country (C) in the six major ITES syndromes, arranged in ascending median age at onset. In (C), dark grey bars in the ‘Asia’ category represent Japan. Reported cases of AESD, ANE, FIRES, and MERS have increased substantially in recent years. Syndromes showed distinct age and geographic distributions. ASEM mainly affected infants; AESD, HHE, and ANE mainly affected preschool children; and FIRES and MERS mainly affected older children and adults. AESD was predominantly reported from Japan, while ANE and FIRES were more globally distributed. ASEM = acute shock with encephalopathy and multiorgan failure, AESD = acute encephalopathy with biphasic seizures and late reduced diffusion, HHE = hemiconvulsion-hemiplegia-epilepsy syndrome, ANE = acute necrotising encephalopathy, FIRES = febrile infection–related epilepsy syndrome, MERS = mild encephalopathy with reversible splenial lesion.

Data were extracted for 2042 patients from 678 studies (Fig. 1). Central review excluded duplicates (n = 42), cases without diagnosis of a specific ITES syndrome (n = 48) and overlap syndromes (n = 6) (Supplementary Figure S1B). A total of 1946 cases (from 656 studies) were analysed including 562 FIRES (155 studies), 422 MERS (204 studies), 414 ANE (140 studies), 217 AESD (64 studies), 164 ASEM (49 studies) and 95 HHE (40 studies). The other syndromes (ALERD [n = 27], AIEF [n = 21], AFCE [n = 15] and AHNE [n = 9]) with <50 patients each were excluded from subgroup comparisons. The updated search after data extraction cutoff identified a further 658 cases from 171 studies that could potentially have been eligible for inclusion (Supplementary Figure S1B footnote). ASEM had the longest publication history, with cases reported steadily over 40 years, while other syndromes showed substantially increased reporting in the last decade (Fig. 2A, Supplementary Figure S2A).

Median age at ITES onset was five years (range 0–81, interquartile range [IQR] 1.8–11, 84% [1635/1946] <18 years). Patients with ASEM were the youngest (median 0.78 [range 0.08–52] years), followed by AESD (1.30 [0.08–15]), HHE (2.00 [0.25–12]), ANE (3.40 [0.10–76]), FIRES (9.00 [0.60–81]), and MERS (9.25 [0–78]) (Fig. 2B, Table 2). Adult onset was most frequent in MERS (33%) (median 31 [range 18–78] years), FIRES (21%) (median 27 [range 18–81] years), and ANE (11%) (median 45 [range 18–76) years), rare in ASEM (1%) and absent in AESD and HHE (Supplementary Figure S2B). Most AESD publications originated from Japan, while studies on other syndromes such as ANE and FIRES were more globally distributed (Fig. 2C). Fifty-four percent (998/1860) of patients were male, without significant difference across syndromes. Pre-existing neurological conditions were generally uncommon, most frequent in AESD (14%) and HHE (13%) (Table 2). Positive family history was noted in ANE (13%).

**Table 2:** Demographics, characteristics of fever and pathogens detected in the six major infection triggered encephalopathy syndromes.

Demographics, characteristics of fever and pathogens detected	ASEM (n = 164)	AESD (n = 217)	HHE (n = 95)	ANE (n = 414)	FIRES (n = 562)	MERS (n = 422)
Age at onset (years) median (range)	0.78 (0.08–52.00)	1.30 (0.08–15.00)	2.00 (0.25–12.00)	3.40 (0.10–76.00)	9.00 (0.60–81.00)	9.25 (0.00–78.00)
Adult (≥18 years) at onset	2 (1%)	0 (0%)	0 (0%)	44 (11%)	118 (21%)	140 (33%)
Male∗	63/131 (48%)	101/217 (47%)	45/95 (47%)	204/413 (49%)	300/510 (59%)	241/422 (57%)
Female∗	68/131 (52%)	116/217 (53%)	50/95 (53%)	209/413 (51%)	210/510 (41%)	181/422 (43%)
Pre-existing neurological problems	17 (10%)	30 (14%)	12 (13%)	19 (5%)	15 (3%)	28 (7%)
Family history of a similar disease^a^	4 (2%)	1 (1%)	0 (0%)	52 (13%)	1 (0%)	13 (3%)
Fever- before neurological onset and afebrile by time of neurological onset	0 (0%)	2 (1%)	2 (2%)	6 (1%)	33 (6%)	20 (5%)
Fever- before neurological onset and still present at neurological onset	67 (41%)	126 (58%)	37 (39%)	133 (32%)	202 (36%)	143 (34%)
Fever- before neurological onset, not specified if still present at neuro onset	40 (24%)	42 (19%)	7 (7%)	126 (30%)	190 (34%)	150 (36%)
Fever- only after neurological onset	2 (1%)	1 (1%)	2 (2%)	0 (0%)	1 (0%)	5 (1%)
Fever- timing of fever not specified	44 (27%)	35 (16%)	43 (45%)	33 (8%)	83 (15%)	44 (10%)
Days between fever onset and neurological onset (median)	1.00	1.00	1.00	2.00	5.00	2.00
Days between fever onset and neurological onset (range)	0.00–7.00	0.00–6.00	0.00–5.00	0.00–21.00	0.00–19.00	0.00–21.00
Maximum recorded temperature (°C) in febrile patients (median)	40.60	39.15	39.00	39.50	39.00	39.00
No microbiological agents detected∗	88/144 (61%)	59/159 (37%)	35/52 (67%)	116/325 (36%)	446/488 (91%)	155/392 (40%)
Influenza virus A∗	9/144 (6%)	21/159 (13%)	3/52 (6%)	85/325 (26%)	1/488 (0%)	34/392 (9%)
Influenza virus B∗	3/144 (2%)	2/159 (1%)	1/52 (2%)	19/325 (6%)	0/488 (0%)	14/392 (4%)
Influenza virus, not otherwise specified (NOS)∗	6/144 (4%)	6/159 (4%)	1/52 (2%)	5/325 (2%)	7/488 (1%)	0/392 (0%)
SARS-CoV-2∗	1/144 (1%)	1/159 (1%)	1/52 (2%)	46/325 (14%)	7/488 (1%)	17/392 (4%)
Human herpes virus 6 (HHV6)∗	3/144 (2%)	35/159 (22%)	3/52 (6%)	12/325 (4%)	0 (0%)	5/392 (1%)
Rotavirus∗	8/144 (6%)	3/159 (2%)	2/52 (4%)	3/325 (1%)	1/488 (0%)	37/392 (9%)
Agent detected in nasopharyngeal aspirate∗	12 (7%)	24 (11%)	5 (5%)	132 (32%)	20 (4%)	55 (13%)
Agent detected in throat∗	1 (1%)	1 (1%)	3 (3%)	2 (1%)	2 (0%)	6 (1%)
Agent detected in serum∗	0 (0%)	9 (4%)	2 (2%)	28 (7%)	8 (1%)	78 (19%)
Agent detected in stool∗	4 (2%)	1 (1%)	2 (2%)	6 (1%)	2 (0%)	32 (8%)
Agent detected in cerebrospinal fluid∗	1 (1%)	7 (3%)	0 (0%)	5 (1%)	1 (0%)	13 (3%)

Fields marked with ∗asterisks were calculated using only available data, and the denominator used is specified in the table.

ASEM = acute shock with encephalopathy and multiorgan failure, AESD = acute encephalopathy with biphasic seizures and late reduced diffusion, HHE = hemiconvulsion-hemiplegia-epilepsy syndrome, ANE = acute necrotising encephalopathy, FIRES = febrile infection–related epilepsy syndrome, MERS = mild encephalopathy with reversible splenial lesion, SARS-CoV-2: severe acute respiratory syndrome Coronavirus 2.

a Family history of a similar disease includes the same condition.

Fever occurred in 97% (1677/1727) patients, almost always preceding and typically still present at neurological symptom onset (Table 2). Median interval between fever and neurological onset was short in all syndromes (<2 days) except FIRES (five days). Median maximum temperatures were 39–40.6 °C, when reported. Microbiological identification varied across syndromes, lowest in FIRES (none identified in 91%). Among patients with micro-organisms detected (n = 689), the commonest were influenza (32%: influenza A 22%, influenza B 6%, unspecified 4%), SARS-CoV-2 (14%), human herpesvirus 6 (8%), rotavirus (8%), and *Mycoplasma pneumoniae* (4%). AESD was linked to HHV-6 (z = 12.87); ANE with influenza A (z = 11.1), SARS-CoV-2 (z = 9.1), and influenza B (z = 4.3); MERS with rotavirus (z = 7.48); and FIRES with absence of microbiological identification (z = 18.2) (all p < 0.001) (Supplementary Table S1). A second agent was identified in 5% (35/689). Most micro-organisms were identified via nasopharyngeal aspirate, with cerebrospinal fluid (CSF) testing negative for micro-organisms when performed.

Regarding acute neurological features, impaired consciousness at presentation was most frequent in ASEM (94%), followed by ANE (73%) and AESD (71%) (Table 3). Seizures were mandatory in FIRES, highly prevalent in HHE (98%) and AESD (92%), and least frequent in MERS (36%). Prolonged seizures ≥30 min were characteristic of FIRES (73%) and also common in AESD (53%) and HHE (45%).

**Table 3:** Symptoms and investigations during the acute illness in the six major infection triggered encephalopathy syndromes.

Symptoms on presentation, investigations	ASEM (n = 164)	AESD (n = 217)	HHE (n = 95)	ANE (n = 414)	FIRES (n = 562)	MERS (n = 422)
Impairment of consciousness	154 (94%)	154 (71%)	39 (41%)	304 (73%)	325 (58%)	268 (64%)
Seizures	140 (85%)	199 (92%)	93 (98%)	232 (56%)	562 (100%)	151 (36%)
Prolonged seizures lasting ≥30 min	22 (13%)	114 (53%)	43 (45%)	24 (6%)	410 (73%)	6 (1%)
Systemic symptoms or complications (shock, haemorrhage, organ failure, etc.)	163 (99%)	8 (4%)	2 (2%)	125 (30%)	70 (13%)	48 (11%)
Shock	158 (96%)	3 (1%)	1 (1%)	85 (21%)	7 (1%)	8 (2%)
Disseminated intravascular coagulation	81 (49%)	1 (1%)	0 (0%)	31 (8%)	2 (0%)	11 (3%)
Thrombocytopaenia (Platelets <150 × 10^9^/L)	108 (66%)	12 (6%)	1 (1%)	111 (27%)	3 (1%)	34 (8%)
Elevated Creatine kinase (CK)	81 (49%)	2 (1%)	2 (2%)	80 (19%)	9 (2%)	17 (4%)
Elevated Aspartate aminotransferase (AST)	132 (80%)	32 (15%)	2 (2%)	227 (55%)	51 (9%)	50 (12%)
Elevated Alanine aminotransferase (ALT)	115 (70%)	26 (12%)	2 (2%)	200 (48%)	45 (8%)	36 (9%)
Elevated lactate dehydrogenase (LDH)	53 (32%)	20 (9%)	1 (1%)	129 (31%)	4 (1%)	25 (6%)
CSF pleocytosis >4 cells/uL∗	5/47 (11%)	6/55 (11%)	4/32 (13%)	46/240 (19%)	183/372 (49%)	101/305 (33%)
Elevated cerebrospinal fluid (CSF) protein >45 mg/dL∗	5/29 (17%)	2/55 (4%)	1/23 (4%)	167/282 (59%)	86/343 (25%)	57/277 (21%)
Abnormal electroencephalogram∗	72/75 (96%)	63/65 (97%)	79/79 (100%)	76/80 (95%)	392/392 (100%)	148/257 (58%)
Genetic tests performed	2 (1%)	21 (10%)	9 (10%)	104 (25%)	133 (24%)	3 (1%)
Genetic tests positive∗	2/2 (100%)	14/21 (67%)	5/9 (56%)	76/104 (73%)	8/133 (6%)	3/3 (100%)

Fields marked with ∗asterisks were calculated using only available data, and the denominator used is specified in the table.

Elevated CK, AST, ALT and LDH defined as above the laboratory's upper limit of normal.

ASEM = acute shock with encephalopathy and multiorgan failure, AESD = acute encephalopathy with biphasic seizures and late reduced diffusion, HHE = hemiconvulsion-hemiplegia-epilepsy syndrome, ANE = acute necrotising encephalopathy, FIRES = febrile infection–related epilepsy syndrome, MERS = mild encephalopathy with reversible splenial lesion.

Systemic complications were nearly universal in ASEM, with shock in 96%, thrombocytopaenia in 66%, disseminated intravascular coagulation (DIC) in 49%, and universally elevated liver transaminases. In ANE there was shock in 21%, thrombocytopaenia in 27%, DIC in 8%, and elevated transaminases in around half. These complications were significantly less frequent in other syndromes.

In other investigations, CSF pleocytosis was present in 32% (354/1106), mostly in FIRES (49%, 183/372) and MERS (33%, 101/305) (Table 3). Elevated CSF protein was present in 31% (326/1036), mostly in ANE (59%, 167/282). Electroencephalogram abnormalities were near universal except in MERS (58%, 148/257). Genetic testing was performed in a minority (up to 25% in ANE and FIRES). Positive genetic findings were most frequent in ANE (18%, 76/414), predominantly pathogenic variants in *RANBP2*; only 1% (8/562) of FIRES had positive findings, including variants in *PCDH19, GABRG2, ALDH7A1*, *TNFRSF13B, NFKB1*, *MT-TF* and *SMC3.*

**Fig. 3:**
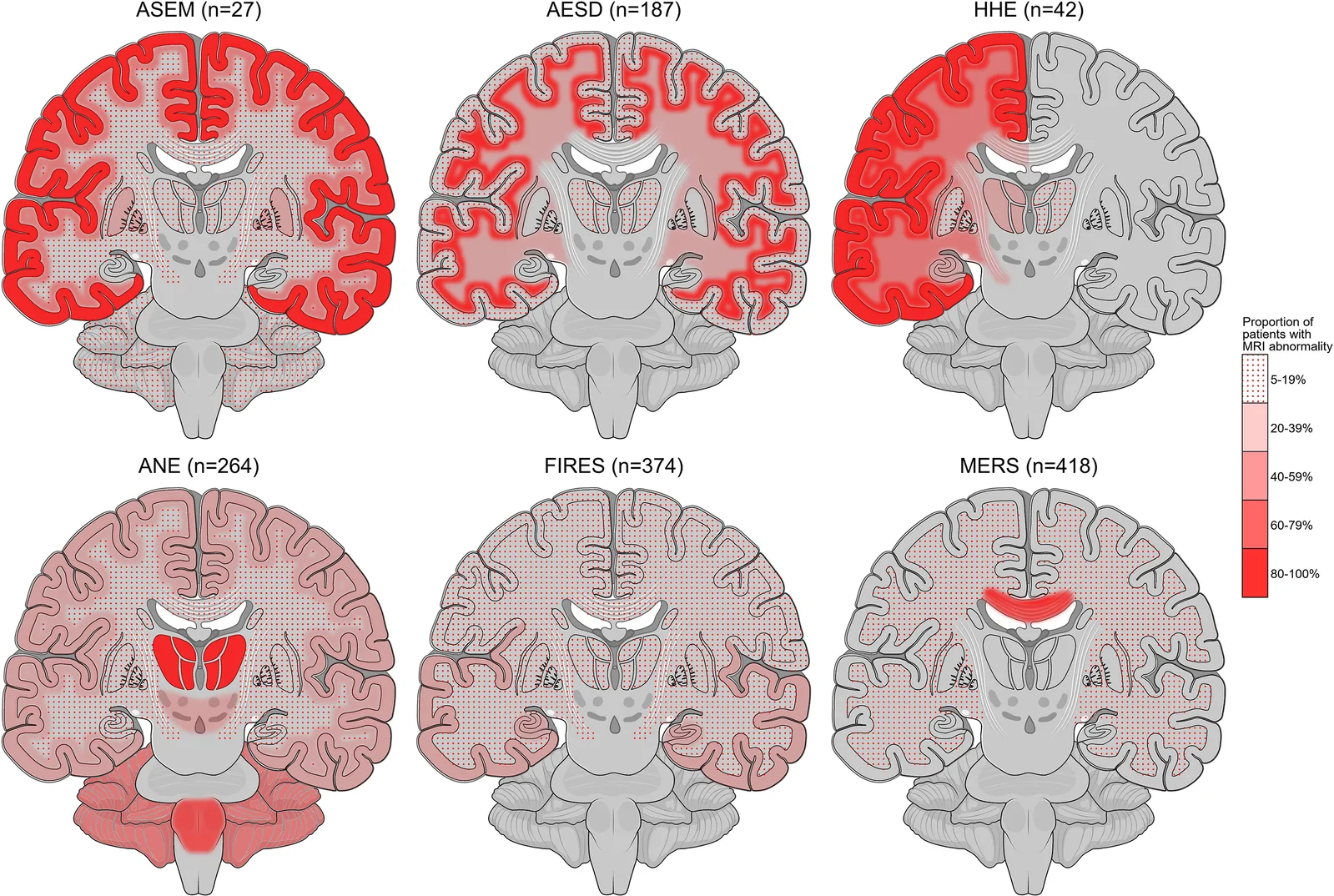
Brain region involvement on acute magnetic resonance imaging (MRI) in the six major infection-triggered encephalopathy syndromes (ITES). Each panel displays a schematic coronal brain section with shading in red indicating the proportion of patients with any MRI abnormality per brain region during the acute illness (first episode) for each major ITES syndrome. MRI abnormalities included restricted diffusion, facilitated diffusion, T2-weighted/fluid-attenuated inversion recovery (FLAIR) hyperintensity, haemorrhagic changes, and cystic/atrophic changes. Regions without shading had <5% involvement. Different ITES syndromes exhibited specific patterns: ANE showed significant thalamic and brainstem involvement, while MERS mainly affected corpus callosum and white matter. ASEM, AESD, and HHE showed variable patterns of white matter (especially subcortical) and cortical grey matter involvement. Abnormalities in FIRES were most frequent in temporal and insular cortices and hippocampus. Regions shown: cortical grey matter, subcortical white matter, deep white matter, corpus callosum, internal capsule, claustrum, caudate, putamen, globus pallidus, thalamus, hippocampus, midbrain, brainstem, and cerebellum. For FIRES only, cortical grey matter is subdivided into frontal lobe, temporal lobe, and insula to reflect the more granular data available for this condition. For HHE only, laterality is depicted: the left side shows overall involvement (unilateral or bilateral), while the right side shows the proportion with bilateral involvement specifically. The number of patients with available data for each syndrome is shown in parentheses. ASEM = acute shock with encephalopathy and multiorgan failure, AESD = acute encephalopathy with biphasic seizures and late reduced diffusion, HHE = hemiconvulsion-hemiplegia-epilepsy syndrome, ANE = acute necrotising encephalopathy, FIRES = febrile infection–related epilepsy syndrome, MERS = mild encephalopathy with reversible splenial lesion.

Regarding acute neuroimaging, median time from neurological onset to first neuroimaging was 2 days, shortest for ASEM and ANE (1 day) and longest for FIRES (3 days) (Table 4). First neuroimaging was computed tomography (CT) in 358 patients (abnormal in 171, 48%) and MRI in 1079 (abnormal in 868, 80%). First CT was most frequently abnormal in ANE (76%, 88/116), least in FIRES (2%, 1/46) and MERS (0%). First MRI was most frequently abnormal in ANE (99%, 160/162) and MERS (98%, 375/381), least in FIRES (51%, 174/338). Median time from neurological onset to first abnormal neuroimaging was 2 days, longest in FIRES (6 days) and AESD (5 days). Forty percent (226/377) of patients with FIRES had normal MRI throughout the entire acute illness. Different syndromes exhibited specific patterns of regional MRI abnormalities (Fig. 3, Table 4, Supplementary Excel S3 for all details of MRI findings). ANE showed significant thalamic and brainstem involvement, while MERS mainly affected corpus callosum and white matter. ASEM, AESD, and HHE showed variable patterns of white matter (especially subcortical) and cortical grey matter involvement. Findings were bilateral in 93% (989/1058), with the notable exception of HHE (14%, 6/42). By MRI feature (Supplementary Figure S3A–D), restricted diffusion was the most frequent finding (77%, 828/1073), notably less in FIRES (13%, 33/248). 74% (836/1137) had T2/FLAIR hyperintensity, least in AESD (23%, 19/82) and FIRES (53%, 196/369). 17% (61/356) of patients with FIRES had claustrum changes reported. Haemorrhagic changes occurred in 15% (113/778), mostly in ANE (62%, 98/158).

**Table 4:** Acute neuroimaging in the six major infection triggered encephalopathy syndromes.

Neuroimaging	ASEM (n = 164)	AESD (n = 217)	HHE (n = 95)	ANE (n = 414)	FIRES (n = 562)	MERS (n = 422)
First neuroimaging- abnormal CT brain scan	57/107 (53%)	14/34 (41%)	11/20 (55%)	88/116 (76%)	1/46 (2%)	0/35 (0%)
First neuroimaging- abnormal MRI brain scan	4/5 (80%)	90/125 (72%)	65/68 (96%)	160/162 (99%)	174/338 (51%)	375/381 (98%)
Days between onset of neurological symptoms and first neuroimaging (median)	1.00	2.00	2.00	1.00	3.00	2.00
Days between onset of neurological symptoms and first neuroimaging (range)	0.00–13.00	0.00–11.00	0.00–10.00	0.00–60.00	0.00–83.00	0.00–21.00
Days between onset of neurological symptoms and first abnormal neuroimaging median (range)	1.00 (0.00–90.00)	5.00 (0.00–24.00)	3.50 (0.00–10.00)	1.00 (0.00–60.00)	6.00 (0.00–700.00)	2.00 (0.00–21.00)
MRI brain haemorrhagic changes	3/12 (25%)	2/70 (3%)	0/32 (0%)	98/158 (62%)	2/258 (1%)	1/217 (0%)
MRI brain restricted diffusion						
Any brain compartment	20/23 (87%)	**183/187 (98%)**	36/36 (100%)	135/150 (90%)	33/248 (13%)	**380/384 (99%)**
White matter	17/23 (74%)	**182/187 (97%)**	**34/36 (94%)**	86/144 (60%)	7/246 (3%)	**380/384 (99%)**
Corpus callosum	2/12 (17%)	6/99 (6%)	0/30 (0%)	8/124 (6%)	3/243 (1%)	**380/384 (99%)**
Cortical grey matter	19/23 (83%)	22/105 (21%)	**29/34 (85%)**	31/132 (23%)	23/245 (9%)	4/325 (1%)
Thalamus	2/15 (13%)	12/83 (14%)	13/30 (43%)	**129/150 (86%)**	5/242 (2%)	1/323 (0%)
Basal ganglia	**8/17 (47%)**	1/83 (1%)	11/30 (37%)	48/143 (34%)	5/241 (2%)	3/324 (1%)
Brainstem	1/15 (7%)	1/129 (1%)	0/31 (0%)	**78/140 (56%)**	0/242 (0%)	1/323 (0%)
Cerebellum	2/15 (13%)	1/99 (1%)	0/33 (0%)	**61/140 (44%)**	0/242 (0%)	7/321 (2%)
MRI brain T2/FLAIR hyperintensity						
Any brain compartment	19/23 (83%)	19/82 (23%)	33/38 (87%)	**253/260 (97%)**	196/369 (53%)	294/332 (89%)
White matter	15/22 (68%)	16/82 (20%)	30/38 (79%)	143/245 (58%)	30/347 (9%)	**292/332 (88%)**
Corpus callosum	2/14 (14%)	1/81 (1%)	10/35 (29%)	13/212 (6%)	5/344 (1%)	**289/332 (87%)**
Cortical grey matter	16/23 (70%)	12/82 (15%)	**31/38 (82%)**	64/234 (27%)	131/363 (36%)	8/292 (3%)
Basal ganglia	2/15 (13%)	4/81 (5%)	13/35 (37%)	**246/260 (95%)**	24/337 (7%)	1/290 (0%)
Cerebellum	6/18 (33%)	3/81 (4%)	10/36 (28%)	**88/237 (37%)**	25/345 (7%)	4/291 (1%)
Thalamus	1/14 (7%)	1/81 (1%)	0/36 (0%)	**158/247 (64%)**	3/341 (1%)	1/289 (0%)
Brainstem	2/15 (13%)	1/81 (1%)	0/37 (0%)	**113/244 (46%)**	3/340 (1%)	7/287 (2%)

The denominators are presented. Features which are >90% of cases are in bold. If no feature is >90%, the syndrome with the highest percentage is in bold.

ASEM = acute shock with encephalopathy and multiorgan failure, AESD = acute encephalopathy with biphasic seizures and late reduced diffusion, HHE = hemiconvulsion-hemiplegia-epilepsy syndrome, ANE = acute necrotising encephalopathy, FIRES = febrile infection–related epilepsy syndrome, MERS = mild encephalopathy with reversible splenial lesion, CT = computed tomography, MRI = magnetic resonance imaging, FLAIR = fluid-attenuated inversion recovery.

**Fig. 4:**
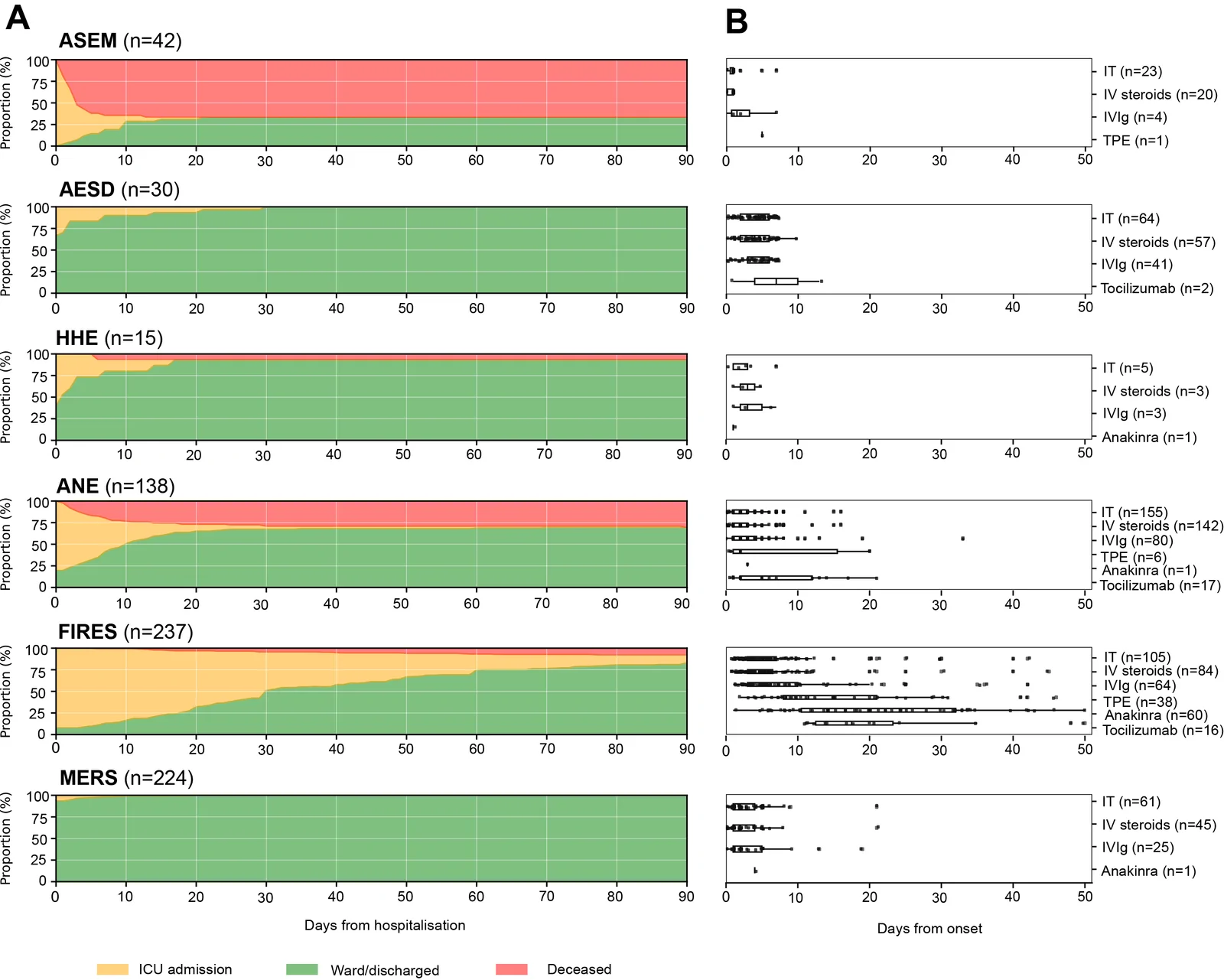
Clinical course and immunotherapy use over time in the six major infection-triggered encephalopathy syndromes (ITES). (A) Proportion of patients with intensive care unit (ICU) admission (yellow), survival outside ICU (green) or death (red) over time for each major ITES syndrome. The number of patients with available data for each syndrome is shown in parentheses. ICU admission was common in FIRES, ANE, and ASEM, often prolonged in FIRES, and rare in MERS, which was generally milder. ASEM and ANE had the highest acute mortality, while FIRES showed more delayed mortality. (B) Timing of immunotherapy use in these syndromes over time. Each dot represents a single patient. The box-and-whisker plot shows the median (centre line), interquartile range (box), and whiskers. Treatment approaches varied between syndromes. ANE and FIRES had the most intensive immunomodulatory treatments, though in FIRES interventions were typically applied sequentially instead of 'upfront'. ASEM = acute shock with encephalopathy and multiorgan failure, AESD = acute encephalopathy with biphasic seizures and late reduced diffusion, HHE = hemiconvulsion-hemiplegia-epilepsy syndrome, ANE = acute necrotising encephalopathy, FIRES = febrile infection–related epilepsy syndrome, MERS = mild encephalopathy with reversible splenial lesion, IT = immunotherapy, IV = intravenous, IVIg = intravenous immunoglobulin.

Onset of the clinical syndrome was typically abrupt with rapid symptom progression, but subsequent course varied by syndrome. Median time from neurological onset to peak severity was 3 days, shortest for ASEM (1 day) and longest for FIRES (7 days) (Supplementary Table S2). Acute mortality was 12% (227/1946), highest for ASEM (50%) and ANE (35%). ICU admission was common in FIRES, ANE and ASEM, often lasting several months in FIRES (Fig. 4A). In contrast, MERS was milder, with few patients experiencing serious impairment or requiring ICU. Hospital stays were longest in FIRES and frequently prolonged in AESD, HHE and ANE; shortest in ASEM (due to mortality) and MERS (due to more benign course).

Immunotherapy in ITES evolved over the years, starting with corticosteroids (1983), followed by IVIG (1995), plasma exchange (1996), rituximab (2012), anakinra (2016), and tocilizumab (2018). Immunotherapy was administered to 61% (1000/1639) patients overall, most frequently and intensively in ANE (82%, 318/387) and FIRES (79%, 378/481) (Fig. 4B, Table 5). Japan reported the highest use of immunotherapies for most ITES syndromes, except for FIRES, where usage was greatest in the United States (distribution by country in Supplementary Excel S2). Median time from neurological onset to first immunotherapy was 3 days, shortest in ASEM (1 day) and longest in AESD (4.5 days) and FIRES (5 days) (Fig. 4B). Corticosteroids were used in 54% (887/1634), most frequently in ANE (78%, 300/386); intravenous immunoglobulin (IVIg) in 38% (620/1618), most frequently in FIRES (61%, 287/470); and plasma exchange in 9% (140/1600), most frequently in FIRES (23%, 107/465) (Table 5). Targeted biologics were used mainly in FIRES (anakinra 16% [74/464], rituximab 6% [28/457], tocilizumab 5% [22/463]) and ANE (tocilizumab 6%, 22/373). Immunotherapy in FIRES was typically sequential rather than ‘upfront’ (Fig. 4B). Ketogenic diet was used predominantly in FIRES (29%, 131/445) and therapeutic hypothermia for neuroprotection in AESD (46%, 41/90) and ASEM (16%, 11/68).

**Table 5:** Treatments in the six major infection triggered encephalopathy syndromes.

Immunotherapy	ASEM (n = 164)	AESD (n = 217)	HHE (n = 95)	ANE (n = 414)	FIRES (n = 562)	MERS (n = 422)
Any immunotherapy	30/84 (36%)	77/132 (58%)	11/78 (14%)	**318/387 (82%)**	378/481 (79%)	148/407 (36%)
Days between onset of neurological symptoms and first immunotherapy, median (range)	1.00 (0.00–7.00)	4.50 (0.00–7.00)	3.00 (1.00–7.00)	2.00 (0.00–16.00)	5.00 (1.00–42.00)	2.00 (0.00–21.00)
Corticosteroids (IV or oral)	27/84 (32%)	72/132 (55%)	8/78 (10%)	**300/386 (78%)**	316/477 (66%)	128/407 (31%)
Intravenous immunoglobulin	6/84 (7%)	51/132 (39%)	7/78 (9%)	198/386 (51%)	**287/470 (61%)**	60/403 (15%)
Plasma exchange	2/84 (2%)	0/132 (0%)	0/78 (0%)	26/373 (7%)	**107/465 (23%)**	2/403 (0%)
Anakinra	0/84 (0%)	0/131 (0%)	1/78 (1%)	3/372 (1%)	**74/464 (16%)**	1/403 (0%)
Tocilizumab	0/84 (0%)	2/132 (2%)	0/78 (0%)	**22/373 (6%)**	22/463 (5%)	1/403 (0%)
Rituximab	0/84 (0%)	0/132 (0%)	0/78 (0%)	1/373 (0%)	**28/457 (6%)**	0/403 (0%)
Others (e.g., cyclophosphamide, azathioprine, mycophenolate)	0/84 (0%)	0/132 (0%)	0/78 (0%)	0/373 (0%)	**15/457 (3%)**	0/403 (0%)
Others						
Anti-seizure medications	73/98 (74%)	**164/168 (98%)**	**89/90 (99%)**	105/222 (47%)	**496/496 (100%)**	79/372 (21%)
Ketogenic diet	0/74 (0%)	0/109 (0%)	3/86 (3%)	0/172 (0%)	**131/445 (29%)**	0/353 (0%)
Neuroprotection- therapeutic hypothermia	11/68 (16%)	**41/90 (46%)**	0/35 (0%)	19/186 (10%)	21/164 (13%)	1/338 (0%)

The denominators are presented. Features which are >90% of cases are in bold. If no feature is >90%, the syndrome with the highest percentage is in bold.

ASEM = acute shock with encephalopathy and multiorgan failure, AESD = acute encephalopathy with biphasic seizures and late reduced diffusion, HHE = hemiconvulsion-hemiplegia-epilepsy syndrome, ANE = acute necrotising encephalopathy, FIRES = febrile infection–related epilepsy syndrome, MERS = mild encephalopathy with reversible splenial lesion, IV = intravenous.

**Fig. 5:**
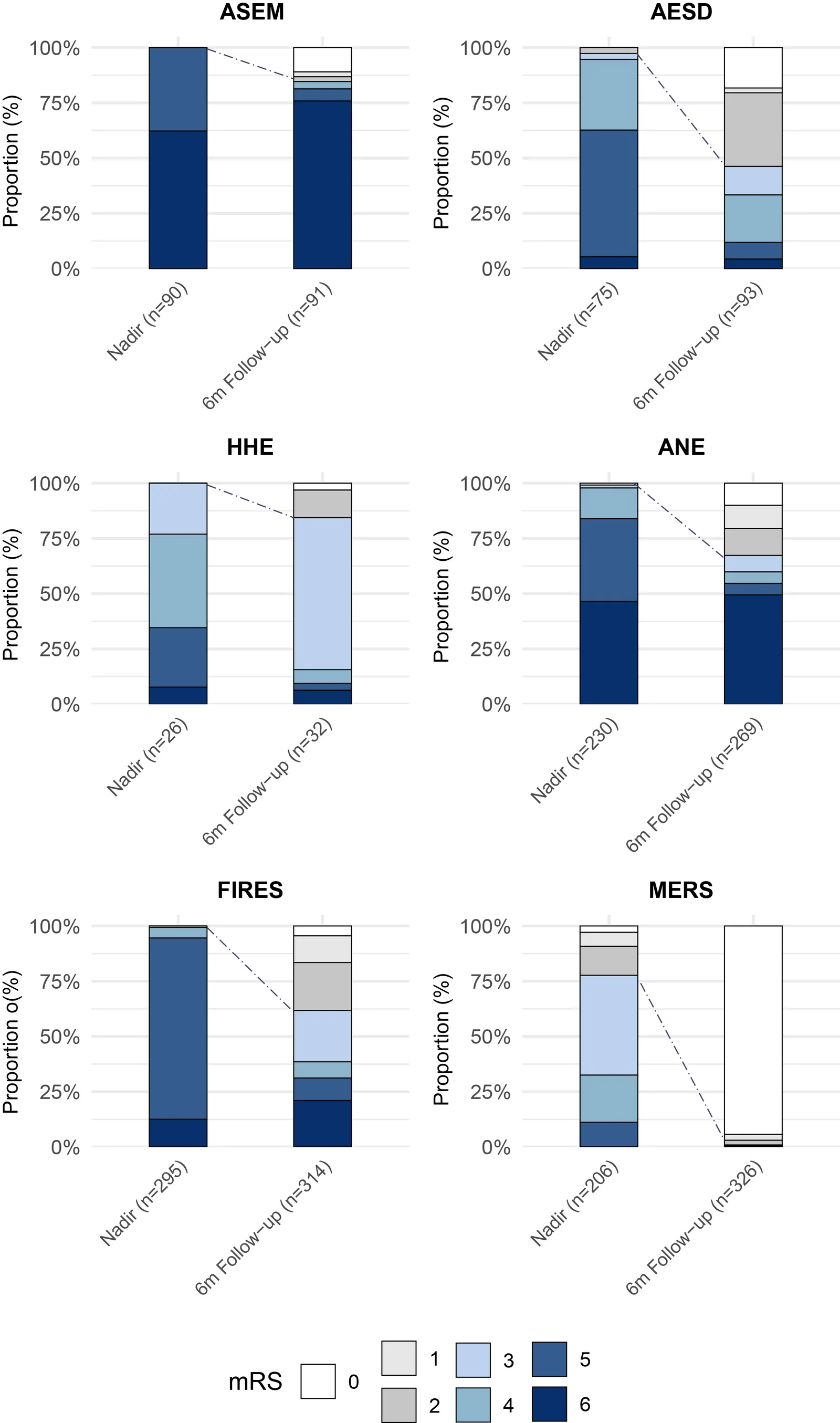
Functional outcomes at nadir and after 6 months follow-up in the six major infection-triggered encephalopathy syndromes (ITES). Modified Rankin Scale (mRS) scores at nadir of the acute illness (first episode) and adjusted mRS after 6 months follow-up for the six major ITES syndromes. Good outcomes (mRS 0–2) are shown in white to light grey, while poorer outcomes (mRS 3–6) are displayed in blue to dark blue. Dotted lines separate mRS 0–2 from 3 to 6. After 6 months follow-up, functional recovery varied between syndromes, with good outcome most frequent in MERS and death most frequent in ASEM and ANE. ASEM = acute shock with encephalopathy and multiorgan failure, AESD = acute encephalopathy with biphasic seizures and late reduced diffusion, HHE = hemiconvulsion-hemiplegia-epilepsy syndrome, ANE = acute necrotising encephalopathy, FIRES = febrile infection–related epilepsy syndrome, MERS = mild encephalopathy with reversible splenial lesion.

The median follow-up was six months, shortest for ASEM (due to mortality) and longest for HHE (Supplementary Table S3). After six months follow-up there was good outcome (mRS 0–2) in 52% (95% CI 50–55; 615/1174): MERS 99% (95% CI 97–100; 323/326), AESD 54% (95% CI 44–64; 50/93), FIRES 38% (95% CI 33–44; 120/314), ANE 33% (95% CI 27–38; 88/269), HHE 16% (95% CI 7–32; 5/32), and ASEM 15% (95% CI 9–24; 14/91) (Fig. 5, Supplementary Figure S4 for follow-up at any time point).

Among survivors, 70% (95% CI 67–73; 615/878) had good outcome (mRS 0–2) at six months: MERS 99% (95% CI 98–100; 323/325), ANE 65% (95% CI 56–72; 88/136), ASEM 64% (95% CI 43–80; 14/22), AESD 56% (95% CI 46–66; 50/89), FIRES 48% (95% CI 42–55; 120/248), HHE 17% (95% CI 7–34; 5/30). Motor deficits were most common in HHE (93%, 86/93), cognitive deficits in HHE (70%, 65/93), FIRES (48%, 238/496) and AESD (46%, 98/213), and epilepsy in FIRES (71%, 351/496) and HHE (79%, 73/93). MERS rarely caused any long-term sequelae (Supplementary Table S3).

Among patients with FIRES, mortality was higher in adults than in children both during the acute phase (16% [14/90] vs 7% [23/332]) and at last follow-up (25% [25/100] vs 14% [41/283]) (Supplementary Excel S4). However, among FIRES survivors, severe cognitive issues were more common in children than in adults (22% [62/282] vs 5% [4/77]). In ANE, mortality at last follow-up was also higher in adults than in children (55% [21/38] vs 38% [112/294]), although acute-phase mortality was similar between the two groups. Clinical outcomes in MERS were comparable in adults and children.

Follow-up MRI (median 52 days from ITES onset, IQR 25–120, Supplementary Figure S5A, Supplementary Excel S3) showed atrophy/cystic changes in 58% (241/417): ASEM 100% (2/2), HHE 99% (67/68), ANE 89% (42/47), AESD 87% (46/53), FIRES 65% (75/115) and MERS 2% (2/125) (Supplementary Figure S5B).

## Discussion

We systematically analysed individual-level published data from 1946 patients with ITES and related syndromes, comparing the six major syndromes. These syndromes share an association with fever or infection and also have other commonalities: they predominantly affect children and produce rapid-onset encephalopathy with characteristic clinico-radiological features. We and others postulate that they have a ‘neuroinflammatory’ substrate arising from a combination of genetic and environmental (infection) factors with secondary excitotoxicity, metabolic failure, and neuroinflammation.^1^^,^^18^ Publications have increased substantially in the last decade indicating increasing recognition, especially since the SARS-CoV-2 pandemic.

We identified several key features. Age was relevant to syndromic vulnerability, with youngest onset in ASEM, AESD, and HHE, followed by ANE, FIRES and MERS. There were no significant differences in sex distribution across the syndromes. Several syndromes showed strong geographic clustering, with AESD predominantly reported from Japan, while ANE and FIRES were more globally distributed. We hypothesise that this may reflect true population-level susceptibility (supported by genetic associations in some syndromes^18^, ^19^, ^20^, ^21^, ^22^, ^23^, ^24^, ^25^, ^26^) but also longstanding recognition of acute encephalopathy syndromes in Japan.^27^ Ascertainment bias cannot be excluded, and AESD may be under-recognised in other regions. Impaired consciousness and seizures were common, with prolonged seizures frequent in FIRES, AESD, and HHE. Apart from MERS, ITES are severe syndromes; ICU admission and mechanical ventilation were common and hospital stays were longest in FIRES.

Fever almost always precedes (and persists at) neurological onset, typically by fewer than two days. ITES is therefore considered ‘para-infectious’ rather than ‘post-infectious’. We identified associations of influenza A with ANE,^28^, ^29^, ^30^ HHV-6 with AESD, and rotavirus with MERS. Most syndromes were associated with respiratory viral infections such as influenza and SARS-CoV-2,^31^^,^^32^ whereas MERS was linked to a broad range of infections (viral, bacterial, and multi-organ origin). By contrast, a specific microbiological trigger was rarely identified in FIRES despite extensive investigation. Given the observational nature of our data, causal relationships between these reported infections and ITES syndromes cannot be established. Further mechanistic studies are needed to better understand these associations. For example, recent work has shown that *RANBP2* regulates influenza viral RNA replication and immune responses, providing understanding into the biological basis of influenza-associated ANE.^33^^,^^34^ It is also important to emphasise that infectious agents were almost never detected in CSF (except occasionally in MERS), reinforcing that ITES should not be considered a central nervous system infection or ‘encephalitis’ but rather an infection-triggered encephalopathy.

Systemic complications were most prominent in ASEM but also seen in ANE; thrombocytopaenia and elevated liver enzymes are important ‘red flags’ for ITES in the context of acute encephalopathy. Some suggest that a peripheral ‘cytokine storm’ could extend into the central nervous system, causing a glial ‘cytokine storm’, though this hypothesis is not definitively proven.^3^^,^^35^, ^36^, ^37^ Routine CSF analysis is usually normal, without the leucocyte infiltration typical of encephalitis, although pleocytosis is relatively common in FIRES, and to a lesser extent MERS. Elevated CSF protein in ANE is hypothesised to reflect protein leak across a disrupted blood–brain barrier due to necrosis and inflammation.^38^ We could not systematically evaluate CSF cytokines due to lack of standardised methods and normative data, but previous research has shown that pro-inflammatory cytokines including interleukin-6 are elevated in ITES.^3^^,^^36^^,^^39^ Other studies report that CSF neopterin, quinolinic acid, and neuron-specific markers are biomarkers of neuroinflammation or neuronal injury and may help distinguish ITES and encephalitis from other causes of new-onset seizures.^35^^,^^40^^,^^41^ Future research exploring blood and central nervous system multi-omics (transcriptomics, proteomics, etc.) will be essential for advancing mechanistic understanding.^42^

Early recognition of ITES is crucial for timely intervention.^17^ Only a minority of patients underwent neuroimaging within the first 24 h of encephalopathy onset, highlighting potential diagnostic delay. Early CT scans showed abnormalities in over half of patients with ANE, ASEM, and HHE, but rarely in FIRES and never in MERS. CT should be considered in the emergency evaluation of patients but has limited sensitivity in some ITES subtypes, highlighting the importance of prompt MRI. Restricted diffusion was the commonest MRI finding, typically bilateral (except HHE) and relatively symmetrical, which can be a useful discriminator from more ‘patchy’ findings in some meningo-encephalitides.^1^ Each syndrome has characteristic MRI patterns including cerebral oedema in ASEM, ‘bright tree appearance’ in AESD,^13^^,^^14^ ‘trilaminar sign’ in ANE,^43^ ‘claustrum sign’ in FIRES,^44^^,^^45^ and reversible splenial lesion in MERS. It is important to note that, in the absence of characteristic ITES neuroimaging findings, many ITES cases remain unclassified. We postulate that these patients are likely common but under-reported or misidentified as ‘encephalitis’.^46^^,^^47^

Genetic testing was rare, performed in only a quarter of ANE and FIRES cases despite increasing evidence for genetic susceptibility in ITES.^18^, ^19^, ^20^, ^21^, ^22^, ^23^, ^24^ In ANE, the genes *RANBP2, RNH1,* and *DBR1* are associated with familial and recurrent ANE.^22^, ^23^, ^24^, ^25^ Additionally, specific human leucocyte antigen genotypes have also been found to confer susceptibility to the aberrant immune responses in ANE.^26^ Due to the rarity of these syndromes and current lack of a systematic approach to genetic testing, clinically significant genetic variants may be missed. Future diagnostic strategies should incorporate recommendations for genetic testing to assess and improve understanding of genetic factors associated with these syndromes. However, based on our understanding, highly penetrant monogenic disorders are rare in ITES, and positive genetic findings in sporadic ANE are rare.^18^ We hypothesise that susceptibility to ITES more commonly involves ‘vulnerability genes' interacting with environmental triggers, rather than single-gene disorders.^18^

Immunotherapy varied across syndromes, with corticosteroids the most common first-line treatment.^48^, ^49^, ^50^ ANE and FIRES received the most intensive regimens including targeted biologics such as cytokine receptor blockers (anakinra, tocilizumab). In ANE, immunotherapy was typically administered within the first ten days; there is emerging evidence that early interleukin-6 (IL6)-receptor blockade with tocilizumab may improve outcomes.^49^^,^^51^, ^52^, ^53^, ^54^ In FIRES initial immunotherapy was also typically within the first ten days, but second-line biologics were often deferred till days 10–30. The 2020 FIRES consensus recommended anakinra and tocilizumab as second-line immunotherapy starting within one week of onset.^11^^,^^50^^,^^55^ Diagnostic practices and treatment options have evolved internationally, with greater physician awareness and increased global availability of emerging immunotherapies.^49^^,^^50^^,^^56^ These advances support the shift toward earlier use of targeted treatments and neuroprotective measures for these devastating conditions.^17^^,^^51^^,^^57^, ^58^, ^59^, ^60^, ^61^

At follow-up, patients with MERS had the most favourable recovery, with minimal or no long-term sequelae.^62^ Other syndromes had poor outcomes (mRS 3–6) in half or more patients, with sequelae that varied between syndromes including refractory epilepsy, cognitive deficits, and functional disabilities.^60^^,^^63^^,^^64^ These considerable differences in presentation and outcome highlight that while ITES serves as an umbrella term, disease mechanisms underlying specific syndromes and their heterogeneous patterns of brain involvement remain unclear.^13^^,^^38^^,^^65^, ^66^, ^67^ ITES syndromes are hypothesised to arise from excitotoxicity, as indicated by seizures and presence of restricted diffusion on MRI.^13^ ANE predominantly involves deeper cerebral and infratentorial structures, often with haemorrhagic-necrotic thalamic changes producing the trilaminar sign.^38^^,^^68^^,^^69^ In contrast, up to 40% of FIRES cases lack acute neuroimaging changes; disability likely arises from cortical neuronal injury secondary to underlying pathogenic processes and super-refractory status epilepticus.^55^^,^^70^

This is the largest systematic study comparing ITES syndromes, providing the most comprehensive dataset so far on these rare conditions. Our primary search was inception to 6 March 2024; an updated search to 14 May 2026 identified up to 658 further potentially eligible cases that were enumerated but not incorporated, in order to preserve a fixed analytic dataset. These additional cases were weighted towards FIRES and AESD relative to the analysed cohort, and their inclusion could have altered some of the whole-cohort estimates. Incorporation of these cases is warranted in future updates. The search was limited to PubMed and selected languages, potentially excluding relevant studies indexed elsewhere or published in other languages. Due to the scale and complexity of this dataset, independent screening of records and subsequent double-entry verification were not feasible within the timeframe of this study.

The international consensus definitions for ITES were only published in 2025; therefore, we included all previous terms meeting the core and individual criteria for ITES and related syndromes.^1^ Some degree of diagnostic misclassification may remain due to evolving disease definitions and variability in reporting practices. Pooled meta-analysis of individual-level published data was a strength, but may introduce ascertainment, publication, and reporting biases. As a result, milder cases may be underrepresented and severe or treatment-responsive cases overrepresented, limiting accurate assessment of disease prevalence and clinical spectrum. By including only individual-level published data, we omitted larger aggregated cohorts that might have important observations.

Findings are subject to the inherent limitations of retrospective real-world data, including incomplete reporting, resulting in a substantial proportion of missing data. Additional limitations of this meta-analysis include reliance on treating physicians’ judgement for diagnosis, and heterogeneity in neuroimaging, infectious disease workups, and other investigations. As pathogens detected by nasal swabs may also be present in asymptomatic individuals, their detection does not necessarily establish a causal relationship with ITES. Electroencephalogram reports were insufficiently detailed for analysis in FIRES. Genetic testing lacked standardisation, with a range of different panels or whole exome sequencing being performed. Timing of investigations and interventions was often unreported, limiting analysis of therapeutic effects on outcome. Additionally, certain treatments such as tocilizumab and anakinra have only become widely used in recent years. The mRS scores often had to be estimated from clinical descriptions, introducing potential subjectivity; our inter-rater agreement was excellent for follow-up scores but only fair-to-good for acute severity. As the published reports measured outcomes at different time points, we used 6-month adjusted mRS scores, assuming that functional independence once achieved was maintained over time. However, this approach has limitations because some cases may theoretically continue to improve or deteriorate, even after achieving early functional independence. Future studies should capture more detailed and holistic outcomes than mRS including psychiatric symptoms, quality of life, caregiver burden, and healthcare system impacts.^71^

Due to the heterogeneity of ITES conditions and missing data, we limited the analysis to descriptive statistics and did not assess outcome measures or treatment efficacy using multivariable analysis. We did not perform clustering of results by study or estimate pooled effect sizes or between-study heterogeneity. Given the large number of single-patient reports included, study-level effect estimates and these statistics could not be meaningfully computed. Additionally, no formal assessment of publication bias, study quality, or certainty was performed. We performed available-case analysis, which may introduce selection bias and limit generalisability. Limited AFCE numbers precluded subgroup comparison with ASEM. We did not examine AESD subtypes, including hemispheric AESD and ALERD, for intragroup similarities and differences.^72^ We only included patients diagnosed with specific ITES syndromes and excluded those with infection-triggered encephalopathy lacking distinctive neuroimaging findings. Sex-specific subgroup analyses were not performed but should be considered in future studies. The relatively small number of adult-onset cases reduces confidence in the differences between age groups and may affect the reliability of the results. Thus, only descriptive comparisons on outcomes between children and adults were performed and should be considered exploratory. Future studies with larger adult cohorts and multivariable analyses are needed to better define age-related differences in characteristics. Based on the current data, it is premature and beyond the scope of this meta-analysis to develop a management algorithm. Nevertheless, this represents an important future direction that should be addressed in subsequent studies, likely involving a Delphi based consensus.

ITES are rare but devastating disorders. To the best of our knowledge, this study brings together the largest group of ITES cases so far, providing a descriptive summary of demographics, clinical characteristics, imaging findings, and outcomes by analysing individual patient data from published reports. This observational data is intended to help pediatricians, emergency physicians, and neurologists identify these conditions more effectively. Future multicenter, prospective studies with standardised data collection will help clarify disease progression, refine therapy, and improve outcomes.

## Contributors

All authors were involved in literature search, data curation, data interpretation, directly assessed and verified the data reported in the manuscript. ME and RCD conceptualised the study and study design. ME and MN developed the search terms and proforma. ME and VXH performed data analysis (bioinformatic, statistical analyses), and generated figures. VXH, ME, RCD were involved in writing- original draft. ME and RCD accessed and verified the underlying data. TT, ML, RCD provided supervision for the study. All authors were involved in writing-review & editing and approved the final manuscript. ME and VXH contributed equally to the study and share first authorship.

## Data sharing statement

Data collected for the study will be shared with any experienced investigator on request to russell.dale@sydney.edu.au.

## Declaration of interests

ME reports a research grant from Action Medical Research (GN2835). MN reports travel support to attend EPNS 2025 (Munich), provided by GW. ML reports grants from the Science and Technology Innovation Program of Hunan Province (2025RC4026); the National Institute for Health and Care Research (NIHR204201; Paediatric Clinical Research Facility Lead); Action Medical Research (GN2945, GN2925, GN2835); the Encephalitis Society; the Boston Children's Hospital Research Fund (GENFD0001772273); and GOSH Charity (VC1421). He received consulting fees from Roche, Novartis, and Octapharma (advisory boards) and honoraria speaking engagements. He performs up to 6 medico-legal cases/year and serves as DMC Chair for the AGSRTI trial (NCT04731103) and on the steering committee for a Phase III Panzyga trial (NCT04508530). He holds unpaid leadership roles with the European Paediatric Neurology Society and the James Lind Alliance/BPNA Research Priority Setting Partnership, and a paid role as Associate Editor, European Journal of Paediatric Neurology.

## Appendix A Supplementary data

Supplementary Material

Proforma

Summary

Neuroimaging

Adult vs child
