# Supplementary material for: Infection-triggered encephalopathy syndromes: a meta-analysis of clinical characteristics and outcomes in 1946 cases
Source: eClinicalMedicine. 2026 Aug 26;99:104163. doi: 10.1016/j.eclinm.2026.104163 (PMC13543797; doi:10.1016/j.eclinm.2026.104163)
Supplement: Supplementary file 1 [file mmc1.pdf]

Infection-Triggered Encephalopathy Syndromes: A Meta-Analysis of Clinical Characteristics and Outcomes in 1946 Cases

Supplementary Materials Contents

Supplementary Methods — Page 2-3

Supplementary Table 1: Infection associations across the six major infection-triggered encephalopathy syndromes (ITES) — Page 4

Supplementary Table 2: Disease severity markers in the six major ITES syndromes — Page 5

Supplementary Table 3: Long term outcomes in the six major ITES syndromes — Page 6

Supplementary Figure 1A: ITES and previous diagnostic terms included in systematic search — Page 7

Supplementary Figure 1B: Flow chart of individuals included in meta-analysis (across ten syndromes) and subgroup analysis (across six syndromes) — Page 8

Supplementary Figure 1C: Number of cases extracted per publication — Page 9

Supplementary Figure 2A: Number of patients from ITES papers included in our meta-analysis- by year of publication — Page 10

Supplementary Figure 2B: Age distribution and age categories of patients across six major ITES syndromes — Page 10

Supplementary Figure 3A: Brain region involvement with restricted diffusion on acute MRI in the six major ITES syndromes — Page 11

Supplementary Figure 3B: Brain region involvement with facilitated diffusion on acute MRI in the six major ITES syndromes — Page 12

Supplementary Figure 3C: Brain region involvement with T2/FLAIR hyperintensity on acute MRI in the six major ITES syndromes —Page 13

Supplementary Figure 3D: Brain region involvement with haemorrhagic changes on acute MRI in the six major ITES syndromes —Page 14

Supplementary Figure 4: Modified Rankin Scale (mRS) scores at nadir and at follow-up (any time point) in the six major ITES syndromes — Page 15

Supplementary Figure 5A: Brain region involvement (any abnormality) on follow-up MRI (at any time point after the first episode) in the six major ITES syndromes — Page 16

Supplementary Figure 5B: Brain region involvement with atrophy/cystic changes on follow-up MRI (at any time point after the first episode) in the six major ITES syndromes — Page 17

Supplementary Excel 1: Proforma of all individual data points collected from papers — separate file

Supplementary Excel 2: Summary of all datapoints for the six major ITES syndromes — separate file

Supplementary Excel 3: Summary of all neuroimaging datapoints for the ITES syndromes — separate file

Supplementary Excel 4: Comparison of outcomes between adult and child-onset ITES syndromes — separate file

Supplementary Methods

Data points collected in proforma

1. Demographics: age, sex, country of publication
2. Clinical symptoms at presentation: impairment of consciousness, seizures, movement disorder, sleep disturbance, speech disturbance, ataxia, psychiatric/behaviour symptoms, cognition/attention/memory impairment
3. Severity markers: systemic symptoms or complications including shock, haemorrhagic episode, multiorgan failure, intravascular disseminated coagulation, admission to intensive care (ICU), duration of hospital stay/ICU, worst (nadir) modified Rankin scale (mRS) (scale of 0 to 6)
4. Investigations: electroencephalogram (EEG), cerebrospinal fluid (CSF), full blood count, liver function test, infectious workup, brain computed tomography (CT) and magnetic resonance imaging (MRI) scans. MRI data were captured with syndrome-specific questions, plus additional brain region (26) x MRI feature (5) fields at up to three timepoints. MRI features were restricted diffusion, facilitated diffusion, T2/FLAIR hyperintensity, haemorrhagic changes and cystic/atrophic changes. Acute MRI abnormalities were counted across all scans acquired during the acute hospitalisation.
5. Treatment: the use of immunotherapy including corticosteroids, intravenous immunoglobulin, plasmapheresis, anakinra, tocilizumab, rituximab etc., detailing the dosage and timing of administration, as well as additional interventions such as anti-seizure medications, ketogenic diet, and neuroprotective strategies including therapeutic hypothermia.
6. Clinical outcomes: Our primary outcome was mRS after at least 6 months follow up. When not reported in the original article, mRS score was retrospectively assigned following review of adequate clinical data provided. Patients achieving mRS 0–2 at any time in recovery (good outcome) had this score assigned as their ≥ 6-month outcome, even if follow-up <6 months. For patients with an mRS of 3–6 measured after six months (poor outcome), the score at the latest follow-up was used. Individuals with an mRS of 3–5 recorded before six months of follow-up were excluded. This approach, as previously used,[1] was designed to capture long-term functional outcomes by retaining early favourable scores (0–2) while only including moderate to severe disability (3–6) if measured beyond six months, ensuring a realistic assessment of recovery.
7. Long term symptoms: epilepsy, motor, cognitive/memory, psychiatry/behavioural problems and relapse rate. Relapses were defined as a new disease event or worsening occurring after a period of improvement or plateau lasting at least three months.

Central data verification steps

This included extensive manual checks for logical consistency across data fields, encoding any residual free text into categorical fields, and revisiting the original reports if any discrepancies were identified. Logical consistency was further enforced through systematic programmatic checks (implemented in Python), including branching-logic validation of field dependencies, propagation of negative parent responses to dependent fields, and flagging of out-of-range laboratory values for unit standardization.

Inter-rater agreement on overall data extraction

20 cases were independently re-extracted by a second reviewer, blinded to the original extraction, yielding 20 paired extractions across 503 patient-level fields (free-text fields were excluded) (10,060 pairs of observations). Because each field contributed at most 20 paired observations, per-field estimates are imprecise; statistics were therefore computed per field and averaged across fields:

Field type	Fields (n)	Metric	Value
All	503	Mean concordance on data availability (scorability)	0.89
Categorical	252	Mean raw agreement	0.92
		Mean Cohen's κ (unweighted)	0.51
		Mean PABAK	0.61
Ordinal	7	Mean quadratic-weighted κ	0.89
		Mean raw agreement	0.94
Continuous numeric	46	Mean intraclass correlation coefficent	0.82

Mean concordance on data availability (scorability) across all 503 fields was 89%, indicating that the two reviewers largely agreed on which fields were reportable, including a substantial number that both judged non-scorable. Of the 11% of field-case observations on which they differed, around two-thirds were instances in which one reviewer recorded an explicit negative or normal finding (e.g. "No", "Normal", "Not given") while the other left the field blank, i.e. reflecting a difference in documentation convention. Lower kappa (chance-corrected) values for categorical data reflect the strong skew of many binary fields toward a single response, which depresses kappa despite high observed agreement. Interpretation: κ 0.40–0.75 fair-to-good, >0.75 excellent.[2]

PABAK= prevalence-adjusted bias-adjusted κ,

Inter-rater agreement on Modified Rankin scale (mRS) score

mRS rating	N pairs	Quadratic-weighted κ (95% CI)	Unweighted κ (95% CI)	PABAK	Raw agreement
**Acute phase**	97	0.58 (0.38–0.77)	0.56 (0.42–0.70)	0.74	77.3%
**Follow-up**	115	0.89 (0.80–0.97)	0.72 (0.63–0.81)	0.74	77.4%

N pairs: Ratings both raters scored numerically. Scorability disagreements (one rater scored, the other judged data insufficient; 20/240) were excluded from κ; if these are instead treated as maximal disagreement as a conservative bound, unweighted κ was 0.52 (acute) and 0.68 (follow-up). Interpretation: κ 0.40–0.75 fair-to-good, >0.75 excellent.[2] PABAK addresses the issue of concentration of the acute-phase mRS score at mRS 5.

CI= confidence interval

References

1. Nosadini M, Eyre M, Molteni E, et al. Use and Safety of Immunotherapeutic Management of N-Methyl-d-Aspartate Receptor Antibody Encephalitis: A Meta-analysis. JAMA Neurology 2021; 78(11): 1333–44.
2. Fleiss, J.L., Levin, B. and Paik, M.C., 2013. Statistical methods for rates and proportions. John Wiley & Sons

Supplementary Table 1: Infection associations across the six major infection-triggered encephalopathy syndromes (ITES)

Significant association defined as z-score ≥3.29 or ≤-3.29, p<0·001 (FDR-adjusted, Benjamini–Hochberg). Positive z-scores indicate positive associations; negative z-scores indicate negative associations.

Feature	FDR-adjusted p-value	Syndrome	Standardised residual (z-score)	Significant association
Human herpes virus 6 (HHV-6)	<0.0001	AESD	12.87	*
		HHE	0.8	
		ANE	-0.03	
		ASEM	-1.09	
		MERS	-2.95	
		FIRES	-5.24	*
Influenza A	<0.0001	ANE	11.14	*
		AESD	1.52	
		MERS	-0.87	
		HHE	-1	
		ASEM	-1.51	
		FIRES	-8.6	*
Influenza B	<0.0001	ANE	4.34	*
		MERS	1.57	
		HHE	-0.27	
		ASEM	-0.34	
		AESD	-1.06	
		FIRES	-4.27	*
Influenza (unspecified)	0·004	ASEM	2.57	
		AESD	2.3	
		HHE	0.19	
		ANE	-0.1	
		FIRES	-0.36	
		MERS	-2.92	
Rotavirus	<0.0001	MERS	7.48	*
		ASEM	1.44	
		HHE	0.15	
		AESD	-1.15	
		ANE	-2.81	
		FIRES	-4.75	*
SARS-CoV-2	<0.0001	ANE	9.09	*
		MERS	-0.37	
		HHE	-0.96	
		ASEM	-2.38	
		AESD	-2.55	
		FIRES	-4.09	*
No microbiological agent identified	<0.0001	FIRES	18.21	*
		HHE	1.44	
		ASEM	0.89	
		AESD	-5.53	*
		MERS	-8.38	*
		ANE	-8.99	*

ASEM= acute shock with encephalopathy and multiorgan failure, AESD= acute encephalopathy with biphasic seizures and late reduced diffusion, HHE= hemiconvulsion-hemiplegia-epilepsy syndrome, ANE= acute necrotizing encephalopathy, FIRES= febrile infection-related epilepsy syndrome, MERS= mild encephalopathy with reversible splenial lesion, mRS= modified Rankin scale

Supplementary Table 2: Disease severity markers in the six major ITES syndromes

Severity	ASEM (n=164)	AESD (n=217)	HHE (n=95)	ANE (n=414)	FIRES (n=562)	MERS (n=422)
Days between neurological symptom onset and time of worst disease severity, median (range)	1.00 (0.5-240)	5.00 (1-120)	2.00 (1-45)	3.00 (1-360)	7.00 (3-999)	2.00 (1-90)
Nadir mRS- 0	0/111 (0%)	0/134 (0%)	0/42 (0%)	2/302 (1%)	0/422 (0%)	6/214 (3%)
Nadir mRS- 1	0/111 (0%)	0/134 (0%)	0/42 (0%)	0/302 (0%)	0/422 (0%)	13/214 (6%)
Nadir mRS- 2	0/111 (0%)	2/134 (1%)	0/42 (0%)	0/302 (0%)	2/422 (0%)	27/214 (13%)
Nadir mRS- 3	0/111 (0%)	4/134 (3%)	7/42 (17%)	5/302 (2%)	0/422 (0%)	95/214 (44%)
Nadir mRS- 4	2/111 (2%)	24/134 (18%)	13/42 (31%)	37/302 (12%)	14/422 (3%)	45/214 (21%)
Nadir mRS- 5	53/111 (48%)	100/134 (75%)	20/42 (48%)	151/302 (50%)	369/422 (87%)	28/214 (13%)
Nadir mRS- 6	56/111 (50%)	4/134 (3%)	2/42 (5%)	107/302 (35%)	37/422 (9%)	0/214 (0%)
Admission to intensive care	123 (75%)	82 (38%)	23 (24%)	208 (50%)	462 (82%)	37 (9%)
Mechanical ventilation	92 (56%)	57 (26%)	14 (15%)	164 (40%)	366 (65%)	12 (3%)
Days of hospital stay (median)	4.00	20.50	20.00	20.50	63.00	9.00
Days of hospital stay (range)	0.50 – 240.00	10.00 – 120.00	6.00 – 45.00	1.00 – 360.00	3.00 – 999.00	1.00 – 90.00

ITES= infection-triggered encephalopathy syndromes, ASEM= acute shock with encephalopathy and multiorgan failure, AESD= acute encephalopathy with biphasic seizures and late reduced diffusion, HHE= hemiconvulsion-hemiplegia-epilepsy syndrome, ANE= acute necrotizing encephalopathy, FIRES= febrile infection-related epilepsy syndrome, MERS= mild encephalopathy with reversible splenial lesion, mRS= modified Rankin scale

Supplementary Table 3: Long term outcomes in the six major ITES syndromes

**Outcomes**	**ASEM (n=164)**	**AESD (n=217)**	**HHE (n=95)**	**ANE (n=414)**	**FIRES (n=562)**	**MERS (n=422)**
Months of last follow-up (median)	0.85	12.00	35.00	6.00	12.00	1.00
Months of last follow-up (range)	0.00 – 48.00	0.00 – 162.00	0.25 – 660.00	0.00 – 222.00	0.00 – 260.00	0.00 – 120.00
Follow-up mRS- 0 at last follow-up	10/113 (9%)	17/141 (12%)	1/43 (2%)	27/332 (8%)	14/383 (4%)	310/329 (94%)
Follow-up mRS- 1 at last follow-up	2/113 (2%)	2/141 (1%)	0/43 (0%)	28/332 (8%)	38/383 (10%)	7/329 (2%)
Follow-up mRS- 2 at last follow-up	2/113 (2%)	31/141 (22%)	4/43 (9%)	33/332 (10%)	68/383 (18%)	6/329 (2%)
Follow-up mRS- 3 at last follow-up	1/113 (1%)	29/141 (21%)	32/43 (74%)	39/332 (12%)	108/383 (28%)	2/329 (1%)
Follow-up mRS- 4 at last follow-up	12/113 (11%)	26/141 (18%)	3/43 (7%)	44/332 (13%)	36/383 (9%)	0/329 (0%)
Follow-up mRS- 5 at last follow-up	17 /113 (15%)	32/141 (23%)	1/43 (2%)	28/332 (8%)	53/383 (14%)	3/329 (1%)
Follow-up mRS- 6 at last follow-up	69/113 (61%)	4/141 (3%)	2/43 (5%)	133/332 (40%)	66/383 (17%)	1/329 (0%)
Motor problems in survivors at last follow-up	22/95 (23%)	72/213 (34%)	86/93 (93%)	103/281 (37%)	82/496 (17%)	4/421 (1%)
Cognitive/memory problems in survivors at last follow-up	16/95 (17%)	98/213 (46%)	65/93 (70%)	85/281 (30%)	238/496 (48%)	8/421 (2%)
Epilepsy in survivors at last follow-up	12/95 (13%)	67/213 (32%)	73/93 (79%)	29/281 (10%)	351/496 (71%)	2/421 (1%)

ITES= infection-triggered encephalopathy syndromes, ASEM= acute shock with encephalopathy and multiorgan failure, AESD= acute encephalopathy with biphasic seizures and late reduced diffusion, HHE= hemiconvulsion-hemiplegia-epilepsy syndrome, ANE= acute necrotizing encephalopathy, FIRES= febrile infection-related epilepsy syndrome, MERS= mild encephalopathy with reversible splenial lesion, mRS= modified Rankin scale

Supplementary Figure 1A: ITES and previous diagnostic terms included in systematic search

ITES core syndromes	Previous or broader terms that were included in the systematic search
Acute shock with encephalopathy and multiorgan failure (ASEM)	Hemorrhagic shock and encephalopathy syndrome
Acute fulminant cerebral edema (AFCE)	
Acute encephalopathy with biphasic seizures and late reduced diffusion (AESD)	
Acute necrotizing encephalopathy (ANE)	Acute necrotizing encephalopathy of childhood (ANEC)
Mild encephalopathy with reversible splenial lesion (MERS)	Reversible splenial lesion syndrome (RESLES)
**ITES-related syndromes**	
Hemiplegia hemiconvulsion epilepsy syndrome (HHE)	
Febrile infection related epilepsy syndrome (FIRES)	Devastating epilepsy in school aged children (DESC)<br>Acute encephalitis with refractory repetitive partial seizures (AERRPS)<br>New onset refractory status epilepticus (NORSE)
**Others**	
Acute infantile encephalopathy predominantly affecting the frontal lobes (AIEF)<br>Acute leukoencephalopathy with restricted diffusion (ALERD)<br>Acute hemorrhagic necrotizing encephalopathy	

Supplementary Figure 1B: Flow chart of individuals included in meta-analysis (across ten syndromes) and subgroup analysis (across six syndromes)

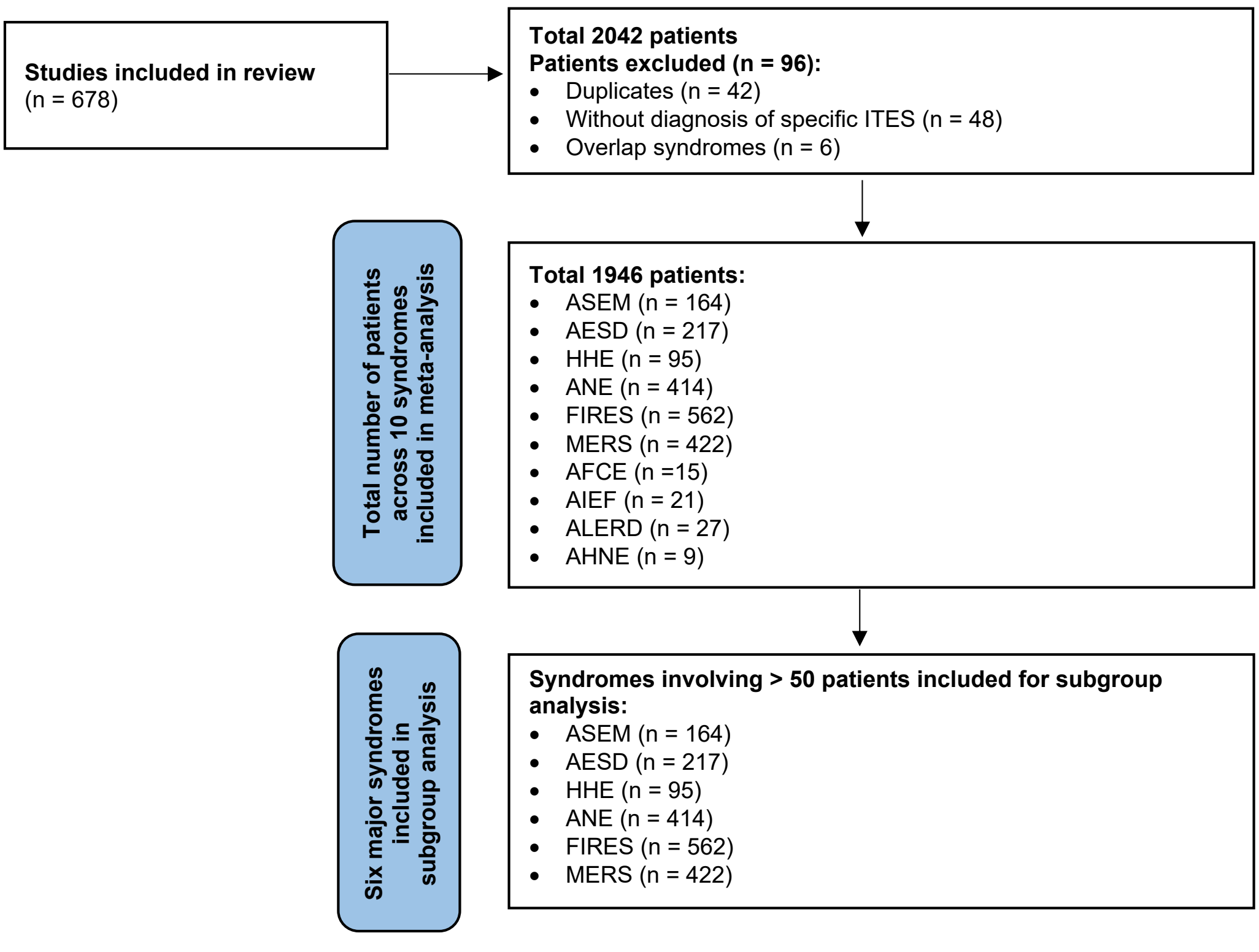

The reasons for exclusion of cases at the central review stage (n=48) were absence of a specific ITES syndrome (e.g. febrile encephalopathy without imaging changes) (n=19), absence of fever/infection trigger (n=8), and presence of an alternative diagnosis (n=21). Alternative diagnoses were encephalitis (n=9), ADEM/demyelination (n=4), metabolic disease (n=4), posterior reversible encephalopathy syndrome (n=3), and hypoxic-ischaemic encephalopathy (n=1).

Updated search (6 March 2024 to 14 May 2026) identified 658 cases (after exclusion of 3 known duplicates) of ITES syndromes with individualised data that were potentially eligible but not extracted or incorporated into the analysis: FIRES (n=295), ANE (n=147), AESD (n=119), MERS (n=53), ALERD (n=18), ASEM (n=13), AFCE (n=8), HHE (n=4) and AHNE (n=1). These counts represent upper limits; following full extraction and central review, a proportion would be expected to be excluded as non-eligible or as further duplicates.

AESD= acute encephalopathy with biphasic seizures and late reduced diffusion; AFCE= acute fulminant cerebral edema; AHNE = acute hemorrhagic necrotizing encephalopathy; AIEF= acute infantile encephalopathy predominantly affecting the frontal lobes; ALERD= acute leukoencephalopathy with restricted diffusion; ANE= acute necrotizing encephalopathy; ASEM= acute shock with encephalopathy and multiorgan failure; FIRES= febrile infection-related epilepsy syndrome; HHE= hemiconvulsion–hemiplegia–epilepsy syndrome; MERS= mild encephalopathy with reversible splenial lesion.

Supplementary Figure 1C: Number of cases extracted per publication

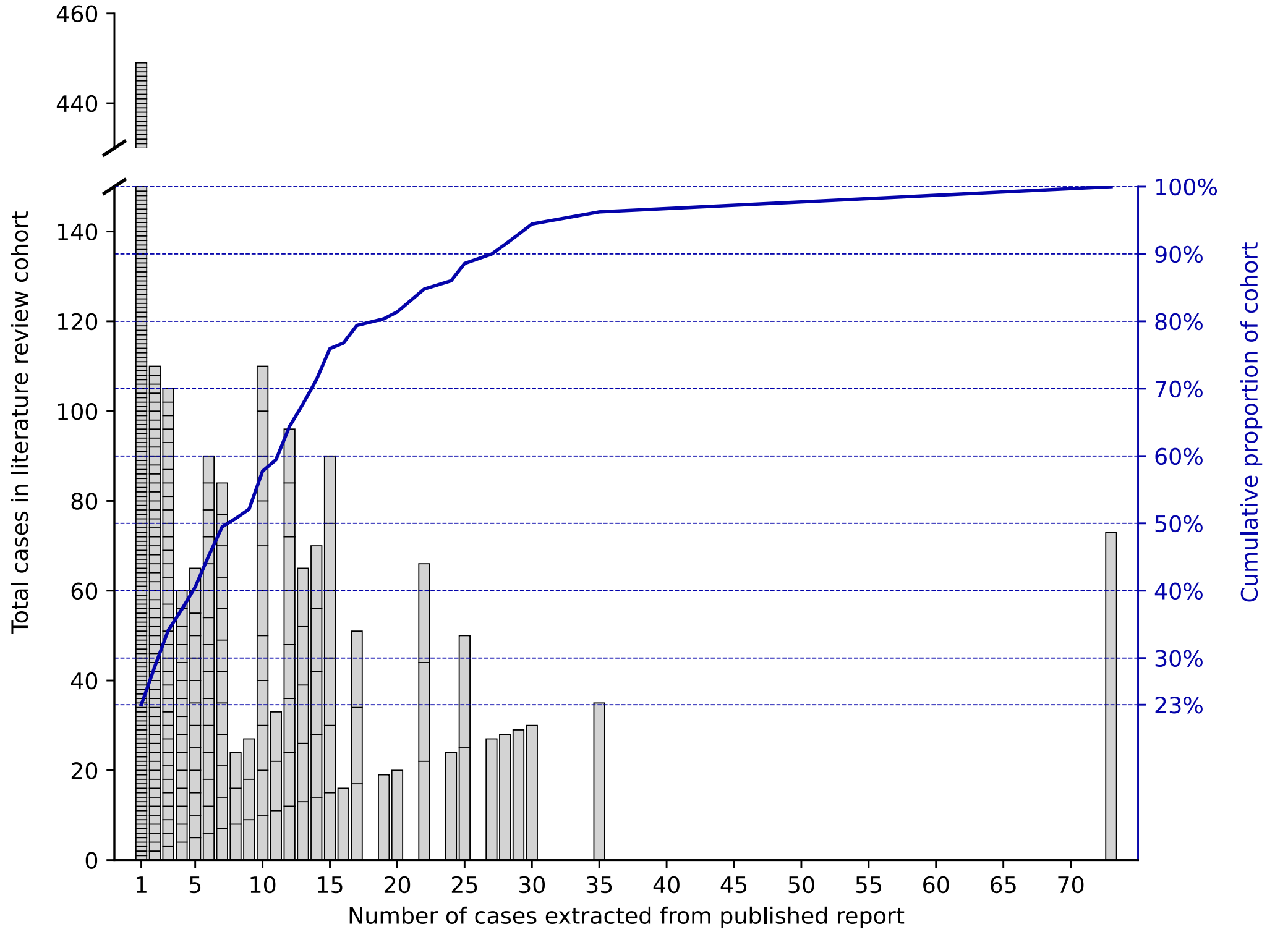

1946 cases were extracted from 656 reports. Each grey box represents one primary report, with its height proportional to the number of cases that report contributed to the cohort. Reports are grouped along the x axis by the number of cases extracted from each, so e.g. the bar at x=1 aggregates all single-case reports (68% of all reports). Bar heights show the total number of cases extracted from reports of that size (left y axis). The blue line shows the cumulative proportion of the total cohort (right y axis) accounted for by reports of a given size or smaller; the majority of cases were extracted from studies reporting 8 cases or less.

Supplementary Figure 2A: Number of patients from ITES papers included in our meta-analysis- by year of publication

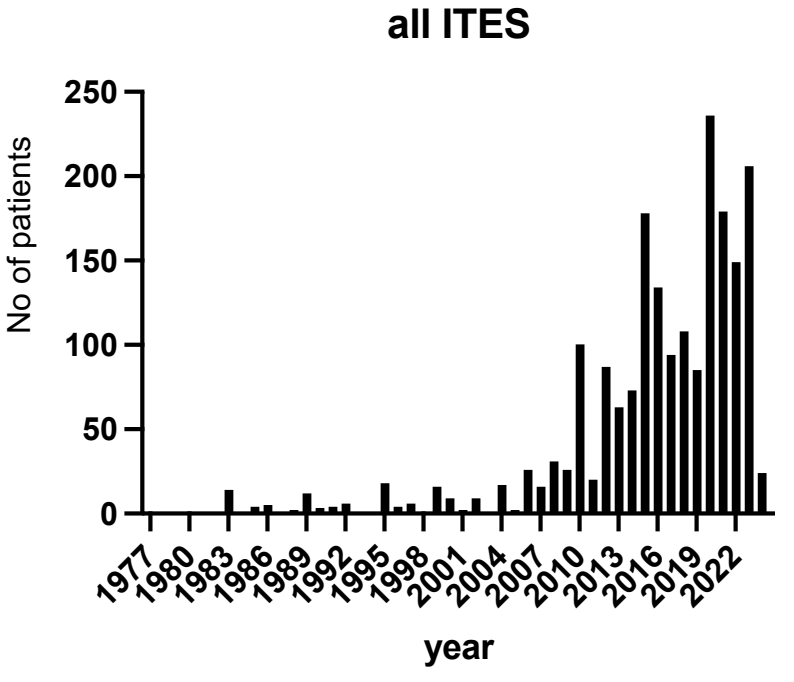

Supplementary Figure 2B: Age distribution and age categories of patients across six major ITES syndromes

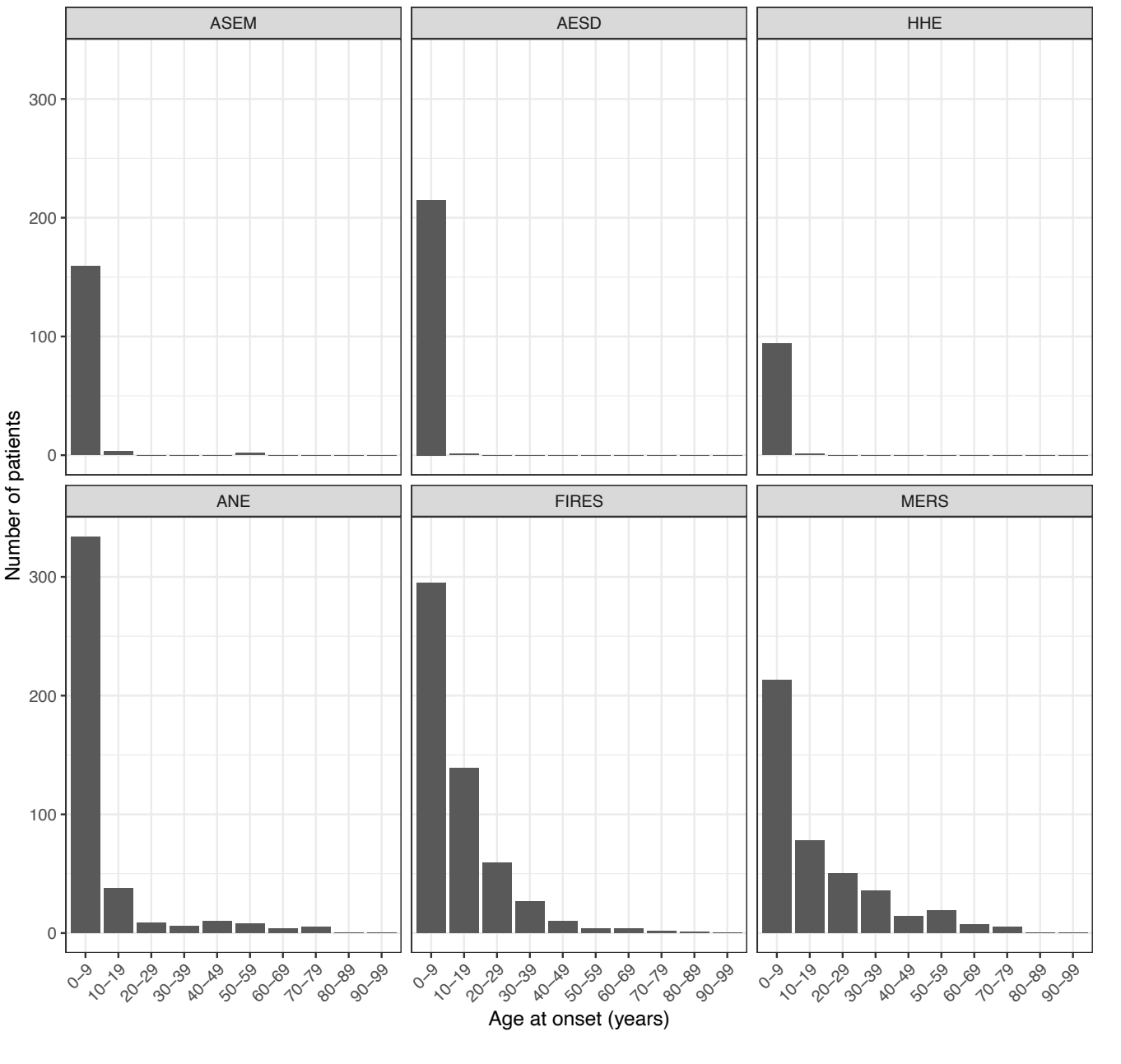

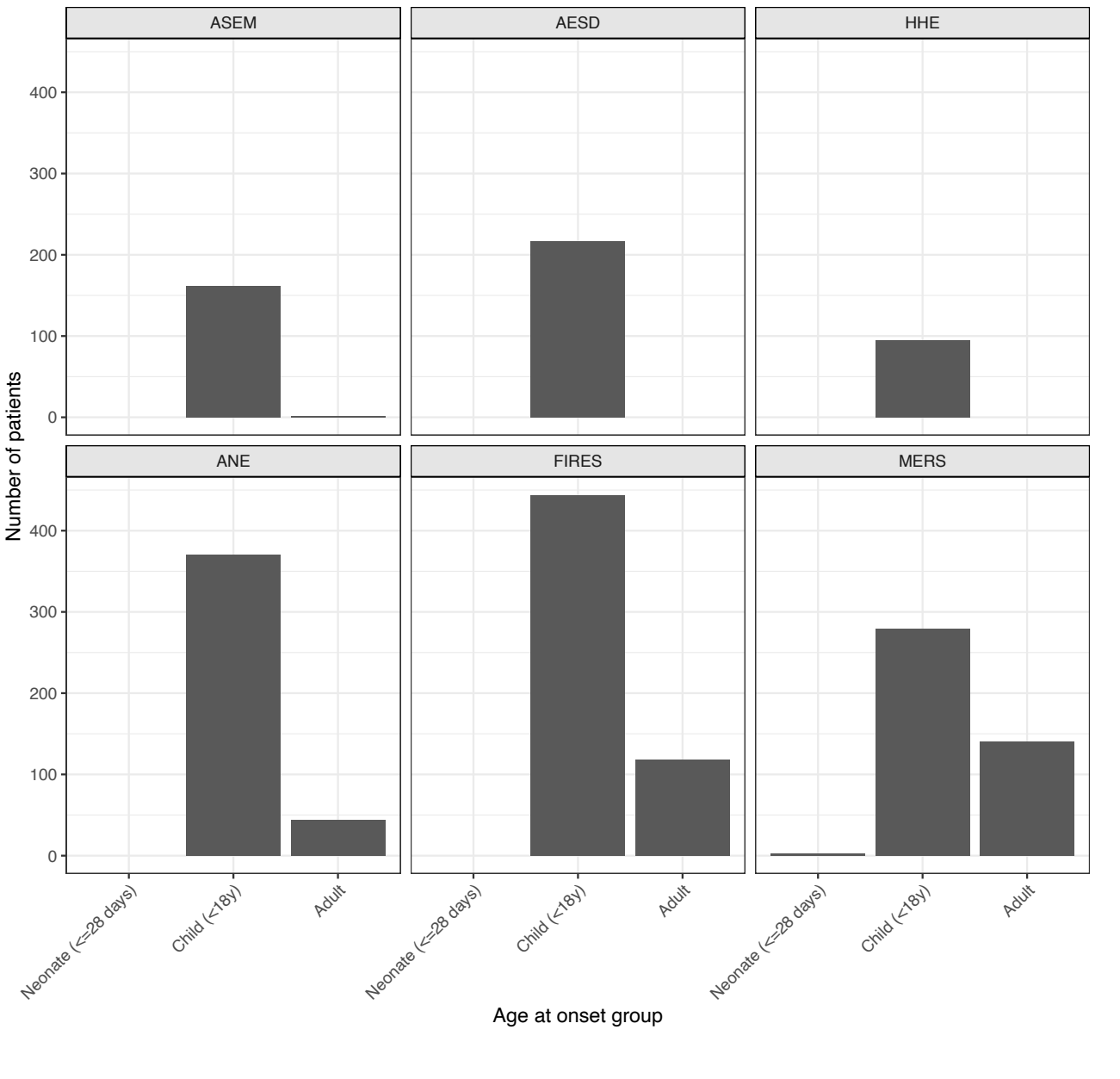

ASEM= acute shock with encephalopathy and multiorgan failure, AESD= acute encephalopathy with biphasic seizures and late reduced diffusion, HHE= hemiconvulsion-hemiplegia-epilepsy syndrome, ANE= acute necrotizing encephalopathy, FIRES= febrile infection-related epilepsy syndrome, MERS= mild encephalopathy with reversible splenial lesion

Supplementary Figure 3A: Brain region involvement with restricted diffusion on acute MRI in the six major ITES syndromes

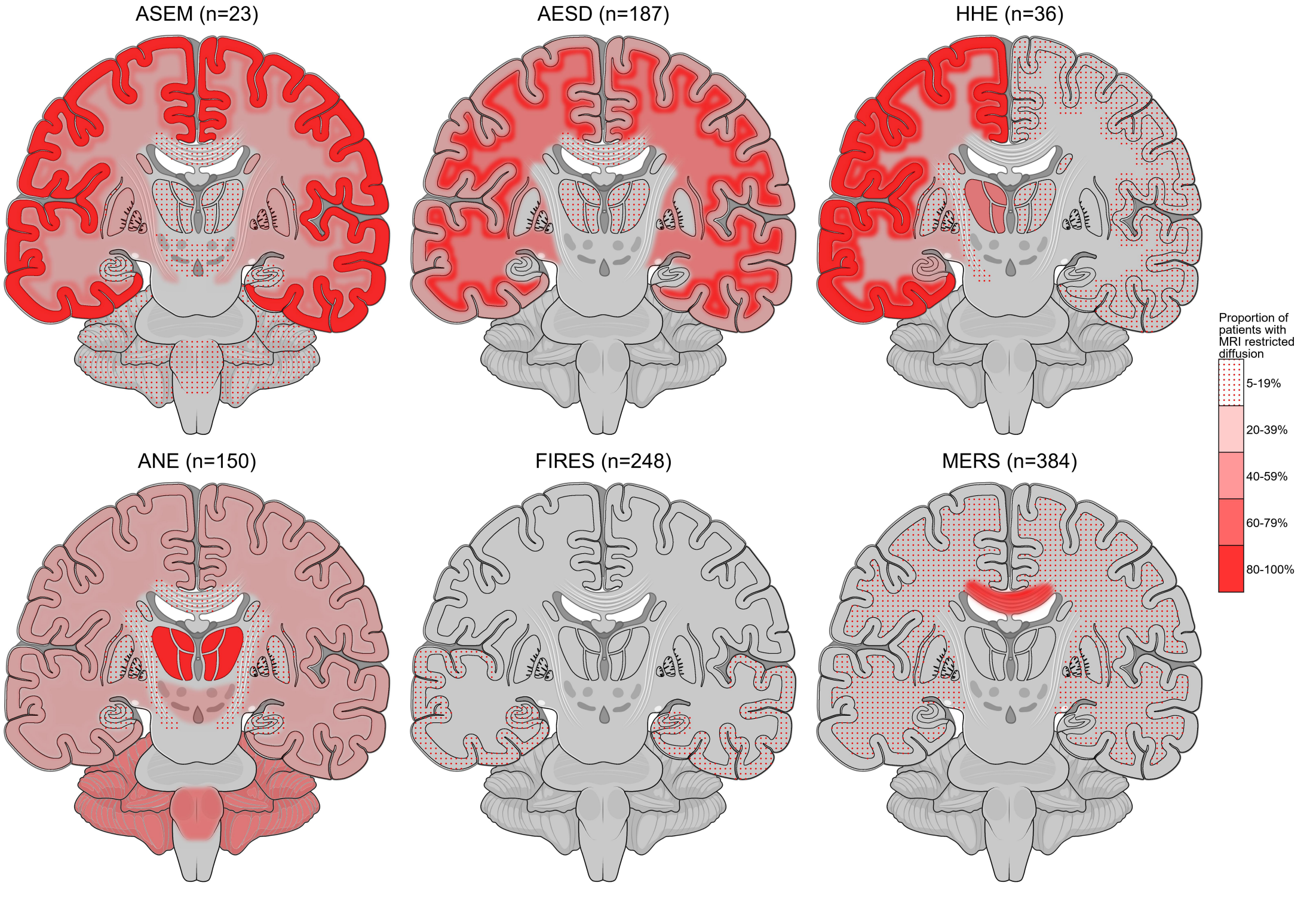

Each panel displays a schematic coronal brain section with shading in red indicating the proportion of patients with restricted diffusion per brain region during the acute illness (first episode) for each major ITES syndrome. Regions without shading had <5% involvement. Regions shown: cortical grey matter, subcortical white matter, deep white matter, corpus callosum, internal capsule, claustrum, caudate, putamen, globus pallidus, thalamus, hippocampus, midbrain, brainstem, and cerebellum. For FIRES only, cortical grey matter is subdivided into frontal lobe, temporal lobe, and insula to reflect the more granular data available for this condition. For HHE only, laterality is depicted: the left side shows overall involvement (unilateral or bilateral), while the right side shows the proportion with bilateral involvement specifically. The number of patients with available data for each syndrome is shown in parentheses.

ASEM= acute shock with encephalopathy and multiorgan failure, AESD= acute encephalopathy with biphasic seizures and late reduced diffusion, HHE= hemiconvulsion-hemiplegia-epilepsy syndrome, ANE= acute necrotizing encephalopathy, FIRES= febrile infection-related epilepsy syndrome, MERS= mild encephalopathy with reversible splenial lesion

Supplementary Figure 3B: Brain region involvement with facilitated diffusion on acute MRI in the six major ITES syndromes

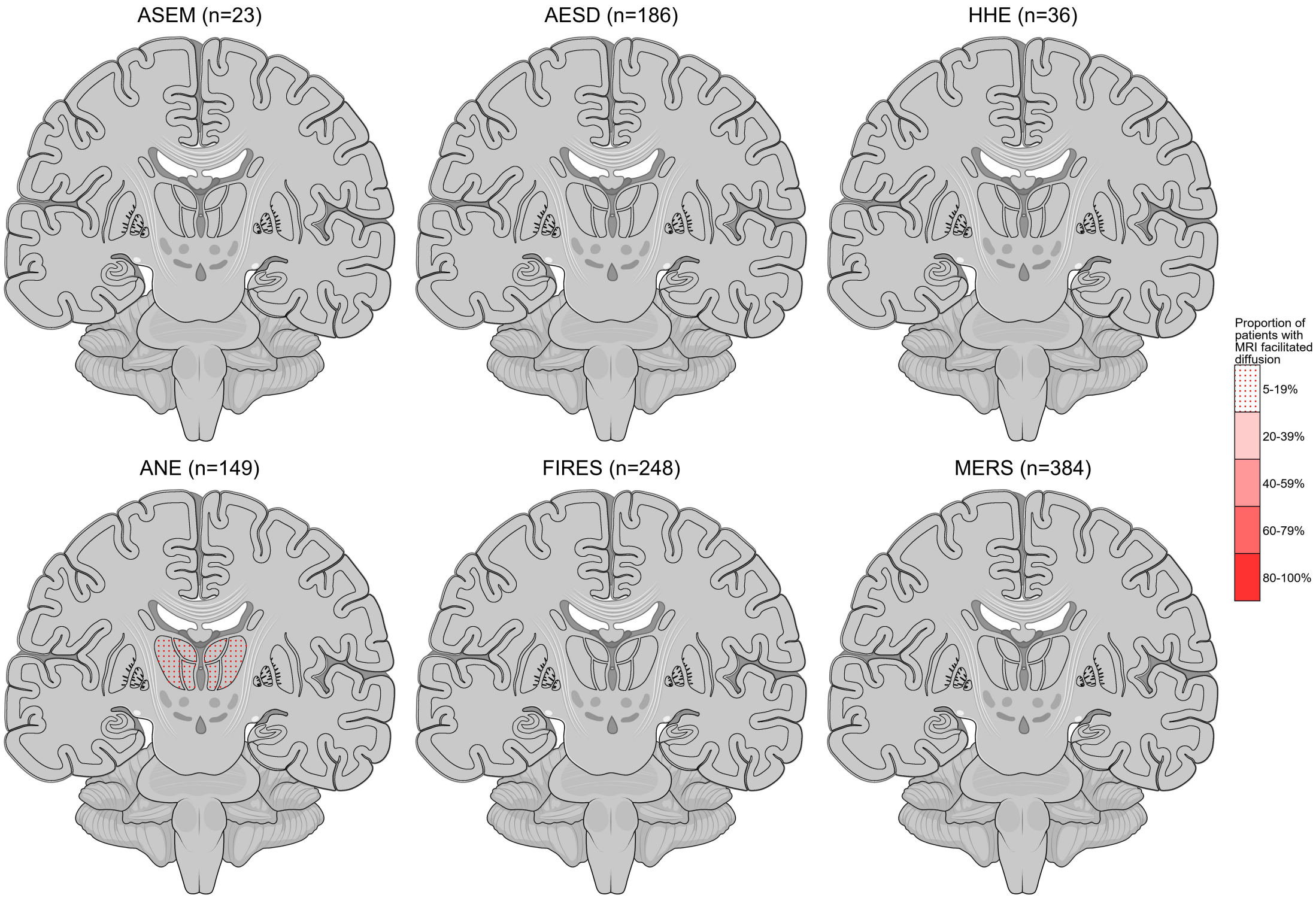

Each panel displays a schematic coronal brain section with shading in red indicating the proportion of patients with facilitated diffusion per brain region during the acute illness (first episode) for each major ITES syndrome. Regions without shading had <5% involvement. Regions shown: cortical grey matter, subcortical white matter, deep white matter, corpus callosum, internal capsule, claustrum, caudate, putamen, globus pallidus, thalamus, hippocampus, midbrain, brainstem, and cerebellum. For FIRES only, cortical grey matter is subdivided into frontal lobe, temporal lobe, and insula to reflect the more granular data available for this condition. For HHE only, laterality is depicted: the left side shows overall involvement (unilateral or bilateral), while the right side shows the proportion with bilateral involvement specifically. The number of patients with available data for each syndrome is shown in parentheses.

ASEM= acute shock with encephalopathy and multiorgan failure, AESD= acute encephalopathy with biphasic seizures and late reduced diffusion, HHE= hemiconvulsion-hemiplegia-epilepsy syndrome, ANE= acute necrotizing encephalopathy, FIRES= febrile infection-related epilepsy syndrome, MERS= mild encephalopathy with reversible splenial lesion

Supplementary Figure 3C: Brain region involvement with T2/FLAIR hyperintensity on acute MRI in the six major ITES syndromes

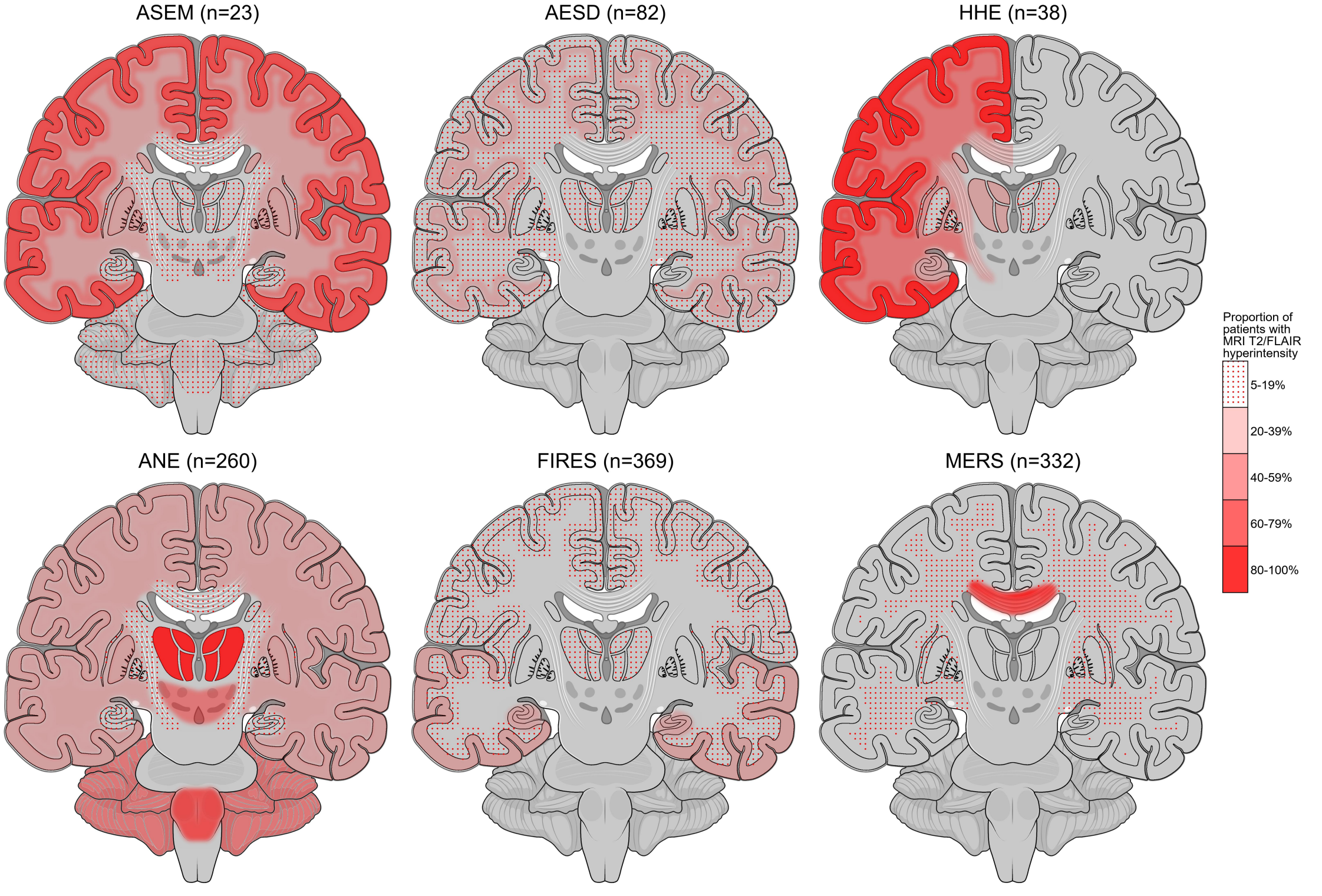

Each panel displays a schematic coronal brain section with shading in red indicating the proportion of patients with T2/FLAIR hyperintensity per brain region during the acute illness (first episode) for each major ITES syndrome. Regions without shading had <5% involvement. Regions shown: cortical grey matter, subcortical white matter, deep white matter, corpus callosum, internal capsule, claustrum, caudate, putamen, globus pallidus, thalamus, hippocampus, midbrain, brainstem, and cerebellum. For FIRES only, cortical grey matter is subdivided into frontal lobe, temporal lobe, and insula to reflect the more granular data available for this condition. For HHE only, laterality is depicted: the left side shows overall involvement (unilateral or bilateral), while the right side shows the proportion with bilateral involvement specifically. The number of patients with available data for each syndrome is shown in parentheses.

ASEM= acute shock with encephalopathy and multiorgan failure, AESD= acute encephalopathy with biphasic seizures and late reduced diffusion, HHE= hemiconvulsion-hemiplegia-epilepsy syndrome, ANE= acute necrotizing encephalopathy, FIRES= febrile infection-related epilepsy syndrome, MERS= mild encephalopathy with reversible splenial lesion

Supplementary Figure 3D: Brain region involvement with haemorrhagic changes on acute MRI in the six major ITES syndromes

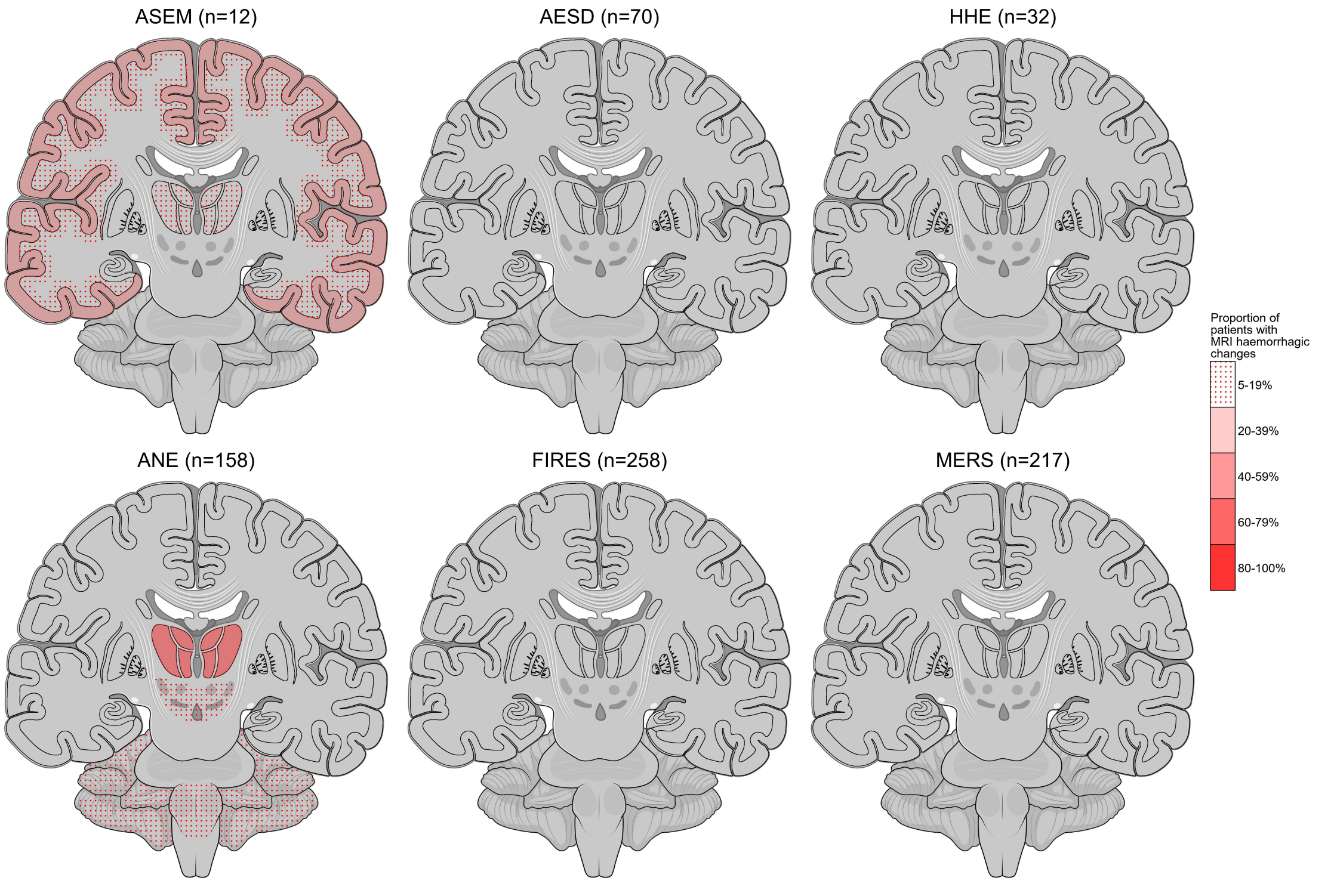

The intensity of the color represents the frequency of these abnormalities in each specific condition. Each panel displays a schematic coronal brain section with shading in red indicating the proportion of patients with haemorrhagic changes per brain region during the acute illness (first episode) for each major ITES syndrome. Regions without shading had <5% involvement. Regions shown: cortical grey matter, subcortical white matter, deep white matter, corpus callosum, internal capsule, claustrum, caudate, putamen, globus pallidus, thalamus, hippocampus, midbrain, brainstem, and cerebellum. For FIRES only, cortical grey matter is subdivided into frontal lobe, temporal lobe, and insula to reflect the more granular data available for this condition. For HHE only, laterality is depicted: the left side shows overall involvement (unilateral or bilateral), while the right side shows the proportion with bilateral involvement specifically. The number of patients with available data for each syndrome is shown in parentheses.

ASEM= acute shock with encephalopathy and multiorgan failure, AESD= acute encephalopathy with biphasic seizures and late reduced diffusion, HHE= hemiconvulsion-hemiplegia-epilepsy syndrome, ANE= acute necrotizing encephalopathy, FIRES= febrile infection-related epilepsy syndrome, MERS= mild encephalopathy with reversible splenial lesion

Supplementary Figure 4: Modified Rankin Scale (mRS) scores at nadir and at follow-up (any time point) in the six major ITES syndromes

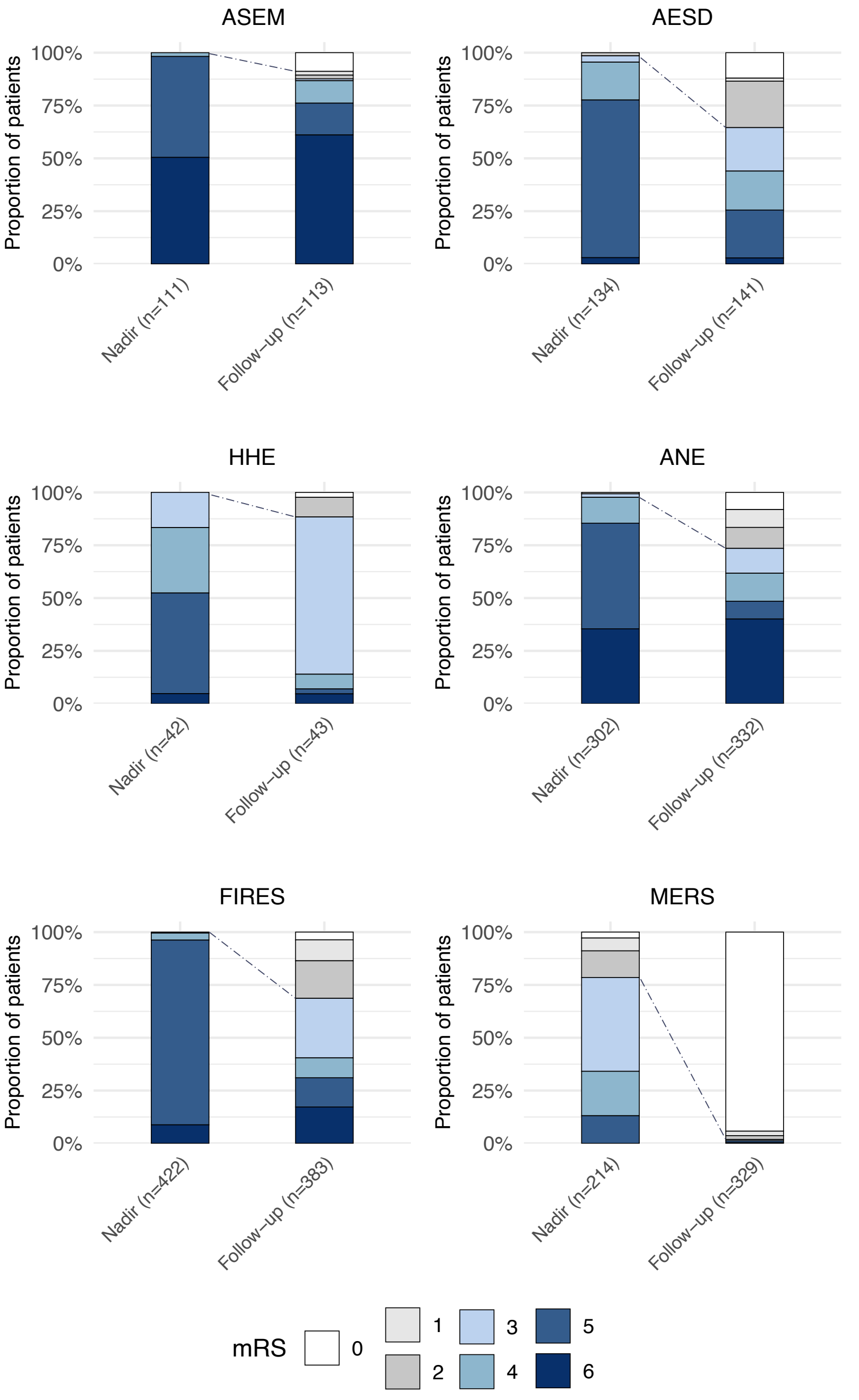

Good outcomes (mRS 0-2) are shown in white to light grey, while poorer outcomes (mRS 3-6) are displayed in blue

ASEM= acute shock with encephalopathy and multiorgan failure, AESD= acute encephalopathy with biphasic seizures and late reduced diffusion, HHE= hemiconvulsion-hemiplegia-epilepsy syndrome, ANE= acute necrotizing encephalopathy, FIRES= febrile infection-related epilepsy syndrome, MERS= mild encephalopathy with reversible splenial lesion

Supplementary Figure 5A: Brain region involvement (any abnormality) on follow-up MRI (at any time point after the first episode) in the six major ITES syndromes

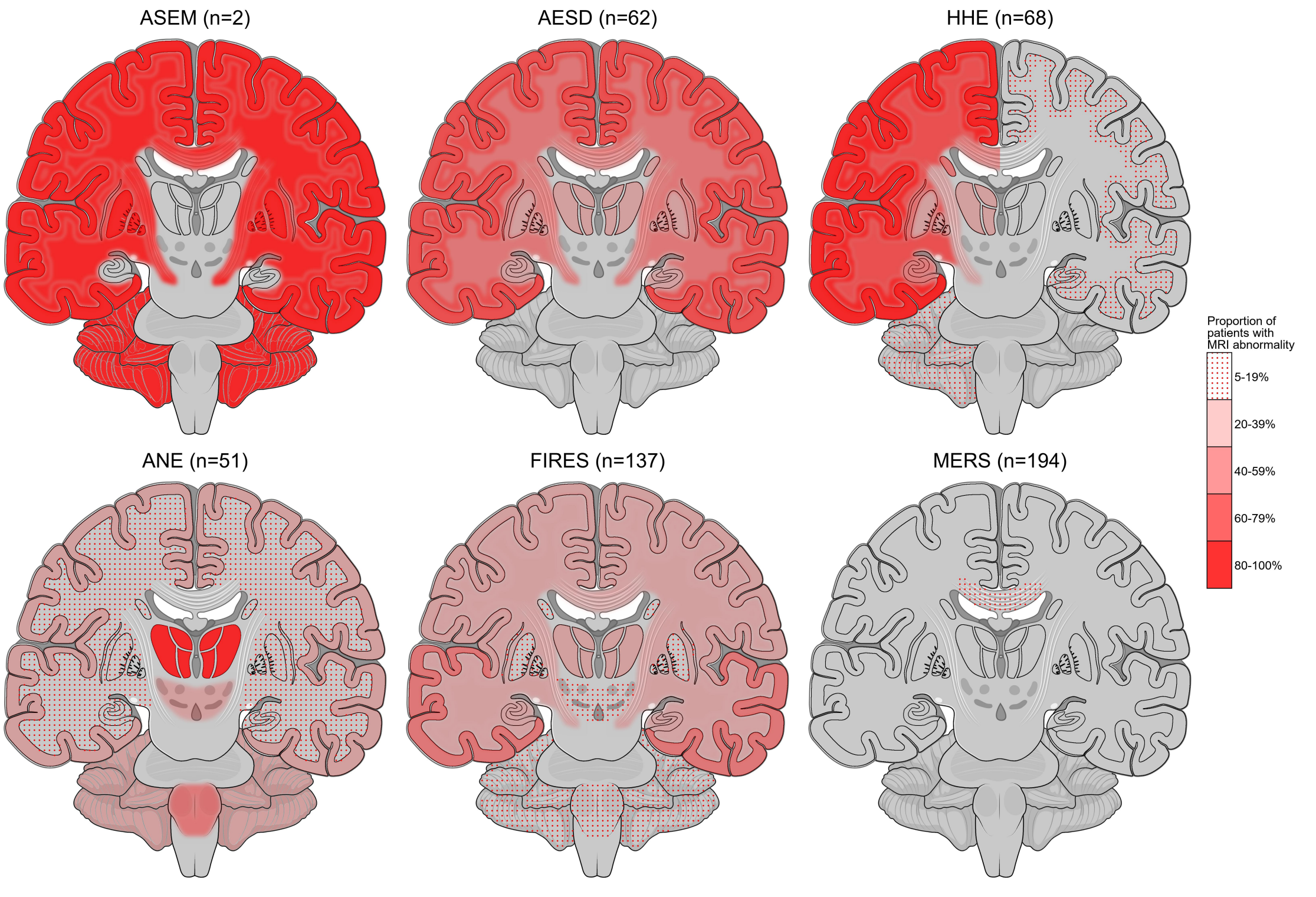

Each panel displays a schematic coronal brain section with shading in red indicating the proportion of patients with any MRI abnormality per brain region on follow-up MRI (at any time point after the first disease episode) for each major ITES syndrome. MRI abnormalities included restricted diffusion, facilitated diffusion, T2-weighted/fluid-attenuated inversion recovery (FLAIR) hyperintensity, haemorrhagic changes, and cystic/atrophic changes. Regions without shading had <5% involvement. For FIRES only, cortical grey matter is subdivided into frontal lobe, temporal lobe, and insula to reflect the more granular data available for this condition. For HHE only, laterality is depicted: the left side shows overall involvement (unilateral or bilateral), while the right side shows the proportion with bilateral involvement specifically. The number of patients with available data for each syndrome is shown in parentheses.

ASEM= acute shock with encephalopathy and multiorgan failure, AESD= acute encephalopathy with biphasic seizures and late reduced diffusion, HHE= hemiconvulsion-hemiplegia-epilepsy syndrome, ANE= acute necrotizing encephalopathy, FIRES= febrile infection-related epilepsy syndrome, MERS= mild encephalopathy with reversible splenial lesion

Supplementary Figure 5B: Brain region involvement with atrophy/cystic changes on follow-up MRI (at any time point after the first episode) in the six major ITES syndromes

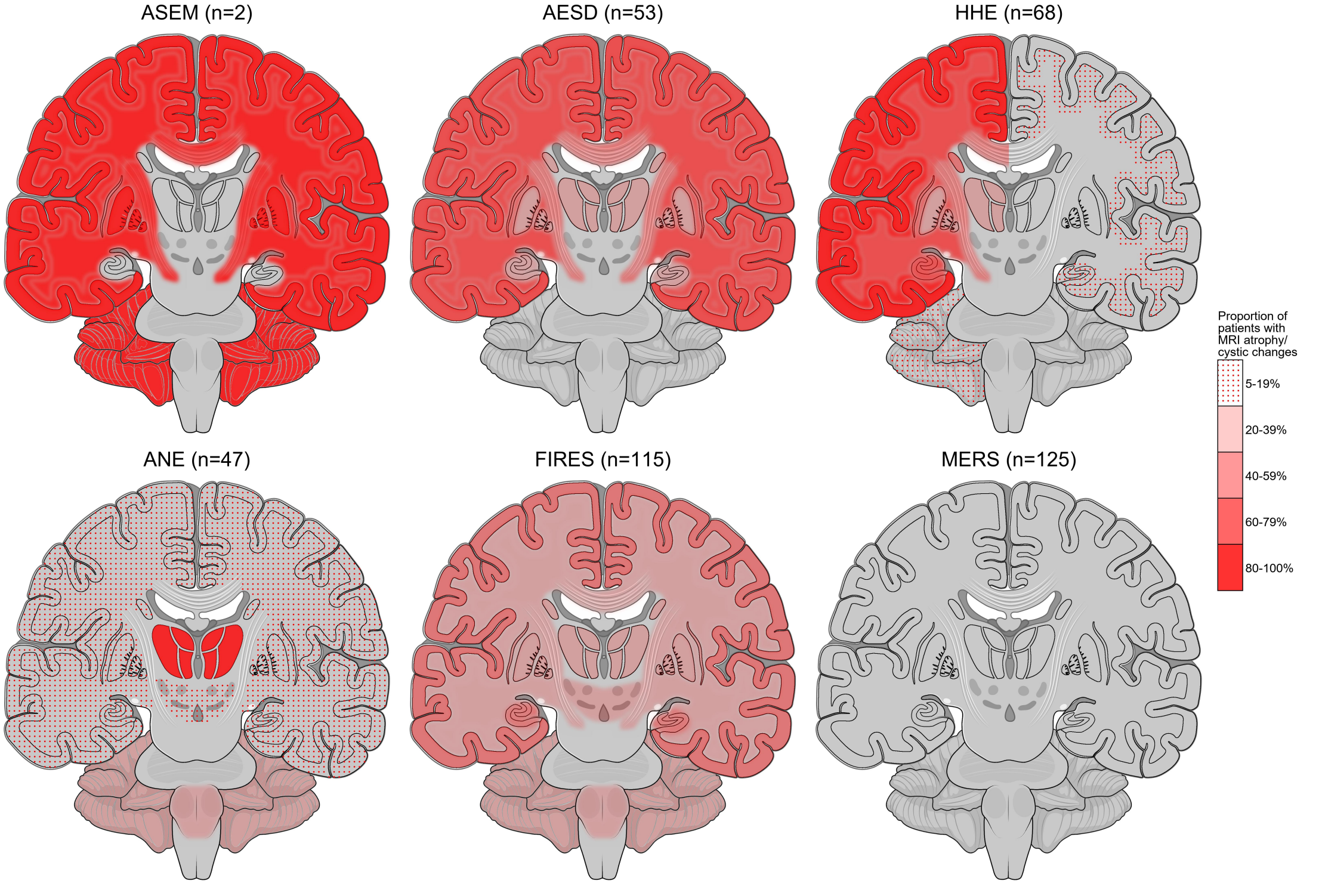

Each panel displays a schematic coronal brain section with shading in red indicating the proportion of patients with atrophy/cystic changes per brain region on follow-up MRI (at any time point after the first disease episode) for each major ITES syndrome. Regions without shading had <5% involvement. For FIRES only, cortical grey matter is subdivided into frontal lobe, temporal lobe, and insula to reflect the more granular data available for this condition. For HHE only, laterality is depicted: the left side shows overall involvement (unilateral or bilateral), while the right side shows the proportion with bilateral involvement specifically. The number of patients with available data for each syndrome is shown in parentheses.

ASEM= acute shock with encephalopathy and multiorgan failure, AESD= acute encephalopathy with biphasic seizures and late reduced diffusion, HHE= hemiconvulsion-hemiplegia-epilepsy syndrome, ANE= acute necrotizing encephalopathy, FIRES= febrile infection-related epilepsy syndrome, MERS= mild encephalopathy with reversible splenial lesion
